# Green Light Therapy for Pain and Pain-Related Psychosocial Outcomes: A Systematic Review of Human Studies

**DOI:** 10.3390/jcm15145710

**Published:** 2026-07-21

**Authors:** Jaclyn Bain, Anirudh Kompella, Ryan Millan, Emily P. Jones, Caroline Sawicki

**Affiliations:** 1 Department of Pediatric Dentistry and Dental Public Health, University of North Carolina, Chapel Hill, NC 27599, USA; jacbain@unc.edu; 2 Adams School of Dentistry, University of North Carolina, Chapel Hill, NC 27599, USA; anirudhk@email.unc.edu (A.K.); rcmillan@live.unc.edu (R.M.); 3 Health Sciences Library, University of North Carolina, Chapel Hill, NC 27599, USA; epjones3@email.unc.edu

**Keywords:** green light therapy, pain management, non-pharmacological analgesia, systematic review, GRADE

## Abstract

**Background/Objectives**: Pain and psychosocial distress are highly prevalent across acute and chronic medical conditions and may negatively affect quality of life, treatment adherence, and clinical outcomes. Green light therapy has emerged as a non-pharmacological intervention that may modulate neural, inflammatory, and sensory pathways involved in nociception and affective processing. This systematic review evaluated the current human evidence on green light therapy for pain, with consideration of pain-related psychosocial outcomes. **Methods**: A systematic search was conducted in PubMed, Scopus, Embase, Cumulative Index to Nursing and Allied Health Literature (CINAHL), and PsycINFO for English-language human interventional studies of green light therapy reporting pain. Records were screened in Covidence by independent reviewers using predefined eligibility criteria. Risk of bias was assessed using the Cochrane Risk of Bias 2 (RoB 2) tool for randomized studies and Risk Of Bias In Non-randomized Studies of Interventions (ROBINS-I) for non-randomized studies. Certainty of evidence was evaluated using the Grading of Recommendations, Assessment, Development and Evaluation (GRADE) framework. **Results**: Of 2040 citations identified, 13 studies met inclusion criteria (9 randomized controlled trials, 4 non-randomized studies; 668 participants). Clinical populations included migraine, dental anxiety, fibromyalgia, autism spectrum disorder, and retinal examination discomfort. Most studies reported findings favoring green light, providing an encouraging evidence base that would be strengthened by future blinded, controlled designs. Certainty of evidence was very low for all outcomes across both comparisons. **Conclusions**: Preliminary evidence suggests green light therapy is a promising and accessible nonpharmacological approach for pain or pain-related psychosocial outcomes but remains insufficient to establish the efficacy of green light therapy. Well-designed, adequately powered, pre-registered, sham-controlled trials with blinded outcome assessment represent a clear and achievable path toward establishing its clinical role.

## 1. Introduction

Pain and psychosocial distress, including anxiety, depression, and stress, are highly prevalent across acute and chronic medical conditions and represent significant barriers to effective treatment delivery and optimal patient outcomes. Chronic pain affects an estimated 20–30% of adults worldwide [1,2], and approximately 40% of adults with chronic pain have clinically significant comorbid depression or anxiety [3], which amplify pain perception, reduce treatment adherence, and diminish quality of life [4,5]. Chronic pain is a multidimensional experience best understood through the biopsychosocial model, which recognizes that pain intensity, psychological distress, and social functioning are interrelated constructs rather than independent outcomes [1]. Pharmacologic therapies remain the primary approach for pain management, but their effectiveness may be limited by variable efficacy, adverse effects, contraindications, cost, and patient preferences [1,6]. In the context of procedural settings (including dental, surgical, and diagnostic procedures), unmanaged pain and anxiety can further compromise treatment acceptance, procedural efficiency, and clinical outcomes, with preoperative anxiety independently predicting higher postoperative pain intensity and opioid consumption [7,8]. These challenges have motivated growing interest in non-pharmacologic adjunctive strategies that can be implemented without disrupting clinical workflow or increasing pharmacologic exposure [9,10].

Among non-pharmacologic approaches, light-based interventions have emerged as a promising modality for pain modulation and stress regulation. In particular, narrow-band green light exposure (wavelength range approximately 495–570 nm) has attracted increasing research attention based on preclinical evidence demonstrating antinociceptive effects through multiple neural pathways [11,12]. Mechanistic studies in animal models have identified several pathways through which green light may exert analgesic effects, including activation of enkephalinergic neurons in the ventrolateral geniculate nucleus (vLGN) projecting to the dorsal raphe nucleus [13], modulation of visual-somatosensory circuits involving the anterior cingulate cortex [14], enhancement of endogenous opioid and endocannabinoid systems [15,16], and reduction in inflammatory mediators [15]. These effects appear to be mediated primarily through cone-dominated retinal pathways rather than through intrinsically photosensitive retinal ganglion cells and require visual exposure rather than cutaneous application [11,13]. Delivery methods in human trials further explore these mechanisms. Ambient green light exposure delivers narrowband green wavelength light (typically 532 nm) directly to the retina over extended periods. In contrast, green-tinted or wavelength filtering lenses modify the spectral composition of ambient light, primarily by attenuating non-green wavelengths that may exacerbate pain or photosensitivity. Red-free (green) light represents another modality, in which green light serves a diagnostic function and exposure is brief. Whether these mechanistically distinct approaches share a common analgesic pathway or operate through independent mechanisms remains an open question.

Clinical investigations of green light therapy have expanded across a range of conditions, including migraine, fibromyalgia, osteoarthritis, procedural pain and anxiety in dental and ophthalmologic settings, and neurodevelopmental populations [12,17]. Delivery methods have varied considerably, encompassing colored goggles, tinted lenses, optical filter spectacles, ambient light-emitting diodes (LED) lamps, and operatory lighting modifications, with exposure parameters ranging from single brief sessions to daily use for several weeks [17,18]. Green light therapy is non-invasive, relatively low-cost, and can be readily integrated into diverse clinical environments, making it an attractive adjunctive option across procedural and chronic pain settings [11,12].

Despite this compelling growing body of research, the existing literature remains heterogeneous with respect to intervention delivery, exposure parameters, clinical populations, and outcome measures. Furthermore, the current evidence has not been systematically synthesized with formal risk of bias and certainty of evidence assessment. The present systematic review was therefore conducted to evaluate the available human evidence on green light therapy for pain and pain-related psychosocial outcomes across clinical contexts, with the aim of characterizing the current state of evidence, identifying methodological limitations, and informing the design of future trials.

## 2. Materials and Methods

### 2.1. Information Sources and Search Strategy

This systematic review is reported in accordance with the Preferred Reporting Items for Systematic Reviews and Meta-Analyses (PRISMA) 2020 statement [19]. The completed PRISMA 2020 checklist is provided in Table S1. The Population, Intervention, Comparison, Outcomes and Study (PICOS) framework guided the development of the research questions, eligibility criteria, and search strategy. Keywords and controlled vocabulary terms were constructed to reflect each PICOS component, including terms related to green light and pain, and human clinical populations (population). These criteria informed database searches (PubMed, Embase, Scopus, Cumulative Index of Nursing and Allied Health Literature (CINAHL), and PsycINFO), as well as the screening and data extraction processes to ensure methodological consistency and reproducibility.

A medical librarian (EJ) developed a comprehensive search strategy to identify potentially relevant publications in PubMed (National Library of Medicine, National Institutes of Health, Bethesda, MD, USA), Scopus (Elsevier, Amsterdam, The Netherlands), Embase (Elsevier, Amsterdam, The Netherlands), CINAHL with Full Text (EBSCOhost, EBSCO Information Services, Ipswich, MA, USA), and PsycINFO (EBSCOhost, EBSCO Information Services, Ipswich, MA, USA). The search strategy used a combination of controlled vocabulary and keywords for green light and pain. Databases were searched from inception to 31 October 2025 and were limited to English language publications only. Full search strategies are available in Appendix A. Additionally, reference lists of included studies and ClinicalTrials.gov (U.S. National Library of Medicine, Bethesda, MD, USA; https://clinicaltrials.gov) were searched to identify ongoing or unpublished studies evaluating green light interventions for pain and psychosocial outcomes. Results were imported into Covidence (Veritas Health Innovation, Melbourne, Australia; https://www.covidence.org) and deduplicated.

This review was registered retrospectively with the Open Science Framework (OSF; Center for Open Science, Charlottesville, VA, USA; https://osf.io (accessed on 31 October 2025)) after data extraction had been completed. To provide transparency regarding the sequence of methodological decisions, the following timeline is documented. Prior to screening: The research question, eligibility criteria, search strategy, and databases to be searched were established by the review team. Prior to data extraction: Screening procedures, data extraction forms, risk of bias assessment tools, and planned narrative synthesis approach were finalized. Prior to analysis: The decision to conduct a narrative synthesis rather than meta-analytic pooling was made a priori based on anticipated clinical and methodological heterogeneity. After data extraction: The protocol was registered with OSF to document the review methods, including the search strategy, eligibility criteria, and planned analyses. The retrospectively registered protocol accurately documents the methods that had been established and followed during the review. No deviations occurred between those methods and the final conduct of the review. OSF registration DOI/URL: 10.17605/OSF.IO/6VXQU.

### 2.2. Eligibility Criteria

#### 2.2.1. Population

Studies were eligible if they enrolled human participants with acute or chronic pain conditions or pain-related psychosocial conditions (e.g., procedural anxiety, sensory distress, or discomfort arising in clinical settings). No restrictions were placed on age, clinical condition, or health status. Studies involving exclusively healthy volunteers with experimentally induced pain (e.g., thermal, mechanical, or electrical pain paradigms) were excluded to prioritize clinical pain conditions and improve applicability to real-world patient populations. This inclusive yet clinically anchored approach is consistent with recommendations for systematic reviews of emerging interventions with limited evidence bases and was adopted for several reasons. First, the hypothesized mechanism of green light analgesia is not condition-specific and may operate across diverse clinical populations. Second, the evidence base for green light therapy in humans remains small, and restricting it to a single population would yield an incomplete picture of the available evidence. Third, the biopsychosocial model of pain recognizes that pain, anxiety, stress, and behavioral responses share overlapping neurobiological substrates, making it appropriate to examine these outcomes across clinical populations where green light has been studied.

#### 2.2.2. Intervention

Eligible interventions included green light therapy delivered through any modality (e.g., light-emitting diodes, filtered lamps, light glasses, ambient room lighting), defined as light within the 495–570 nm wavelength range. Studies were required to isolate green light as a distinct intervention; those evaluating non-specific light therapies, polychromatic light without a defined green component, or photobiomodulation targeting tissue rather than visual pathways were excluded, as these interventions involve distinct biologic mechanisms.

#### 2.2.3. Comparator

Any comparator was permitted, including no treatment, sham/placebo light, alternative wavelength light (e.g., white, red, blue), standard care, or waitlist control.

#### 2.2.4. Outcomes

Studies were required to report quantitative assessment of at least one of the following: pain intensity (e.g., visual analog scale (VAS), numeric rating scale (NRS), or validated pain questionnaires); pain-related psychosocial outcomes (e.g., anxiety, stress, catastrophizing, or quality of life measures); physiological measures related to pain or stress response (e.g., heart rate variability, cortisol levels). Studies lacking extractable outcome data for the above measures were excluded.

#### 2.2.5. Study Design

Only interventional study designs were eligible, including randomized controlled trials and non-randomized controlled trials (e.g., quasi-experimental designs, controlled before-after studies), in order to prioritize evidence capable of evaluating causal effects of green light therapy on pain and pain-related psychosocial outcomes. Observational studies (cohort, case–control, cross-sectional), case reports, case series, narrative reviews, systematic reviews, editorials, commentaries, and conference abstracts without sufficient methodological detail were excluded due to increased susceptibility to confounding and expectancy effects, particularly for subjective outcomes such as pain and anxiety.

#### 2.2.6. Additional Criteria

Studies not available in English were excluded. No restrictions were applied based on publication date or setting. Studies published only as abstracts were excluded if insufficient data were available for quality assessment and data extraction.

### 2.3. Selection Process

Search results were exported to Covidence systematic review software (Veritas Health Innovation, Melbourne, Australia) for deduplication and screening. Two reviewers independently screened titles and abstracts against the predefined eligibility criteria, followed by full-text review of potentially eligible records. Discrepancies at each stage were resolved through discussion; a third reviewer was consulted if consensus could not be reached. Reasons for exclusion at the full-text stage were documented and reported in accordance with PRISMA guidelines.

### 2.4. Data Collection Process

Two reviewers independently extracted data from included studies using a standardized form. Discrepancies were resolved through discussion or consultation with a third reviewer. Variables extracted included: study characteristics (author, year, country, design, registration status, funding); participant characteristics (sample size, age, sex, clinical condition); intervention and comparator characteristics (delivery method, wavelength, intensity, exposure duration and frequency); and outcome data (measurement instrument, assessment time points, effect direction and magnitude including *p*-values, effect sizes, and confidence intervals where reported). When outcome data were reported incompletely, available data were extracted as reported and limitations noted. No imputation of missing summary statistics was performed. Study authors were not contacted to obtain or confirm missing data.

### 2.5. Risk of Bias Assessment

Risk of bias was assessed independently by one reviewer with a second reviewer verifying the assessments. Studies with true randomization (including informal methods such as coin toss) were assessed using the Cochrane Risk of Bias 2 (RoB 2) tool, which evaluates bias across five domains encompassing randomization, deviations from intended interventions, missing outcome data, outcome measurement, and selection of reported results [20]. Each domain was judged as low risk, some concerns, or high risk, and an overall risk of bias judgment was derived according to the RoB 2 algorithm. Several included studies employed non-standard designs (within-subject crossover); thus, crossover trials were assessed using the Rob2 for Crossover Trials tool. The crossover adaptation includes additional signaling questions addressing carryover effects, period-specific biases, and appropriateness of the crossover design for the condition under study. Studies described by their authors as observational were reclassified based on actual methodology when the investigators assigned the intervention.

For non-randomized studies of interventions, the Risk of Bias in Non-Randomized Studies of Interventions (ROBINS-I) tool was applied, which assesses bias across seven domains encompassing confounding, participant selection, intervention classification, deviations from intended interventions, missing data, outcome measurement, and selection of reported results [21]. Each domain was judged as low risk, moderate risk, serious risk, or critical risk, with an overall judgment reflecting the most severe rating across domains.

Any discrepancies between reviewers were resolved through discussion or consultation with a third reviewer. When methodological details were ambiguous, the most conservative interpretation was applied for risk of bias assessment. Results of the risk of bias assessments are presented in figure format and summarized narratively. The potential impact of risk of bias on the certainty of evidence was considered when interpreting the findings of this review.

### 2.6. Certainty of Evidence Assessment

The certainty of evidence for each outcome was assessed using the Grading of Recommendations Assessment, Development and Evaluation (GRADE) framework [22,23,24,25]. For randomized controlled trials, evidence began at high certainty; for non-randomized studies, evidence began at low certainty, consistent with GRADE guidance. When both randomized controlled trials (RCT) and non-randomized studies contributed to the same outcome within a comparison, the evidence was assessed separately by study design and the overall certainty was determined by the body of evidence that provided the most informative basis for the rating, following GRADE guidance for integrating different study designs. All evidence was evaluated for potential downgrading across five domains: risk of bias, inconsistency, indirectness, imprecision, and publication bias [24]. Each domain was rated as not serious (no downgrading), serious (−1 level), or very serious (−2 levels). No domains were considered for upgrading, as the conditions for upgrading (large magnitude of effect, dose–response gradient, or plausible confounding increasing confidence) were not met for any outcome. Risk of bias ratings were informed directly by the RoB 2 and ROBINS-I assessments. Because meta-analysis was not performed, inconsistency was assessed qualitatively. Publication bias was assessed qualitatively given the small number of studies per outcome. Specific justifications for each domain rating are reported alongside the GRADE findings in Section 3.5.

Pain was designated as the critical outcome across all comparisons. Psychosocial outcomes, including anxiety, physiological stress markers, photophobia, opioid consumption, behavioral cooperation, and patient preference, were designated as important outcomes. The GRADE assessment was performed separately for each intervention subgroup (ambient/filtered green light; green-wavelength laser) and, within each subgroup, by comparison type (green light vs. sham/no treatment; green light vs. other wavelengths). Findings are presented with effect estimates described qualitatively rather than as pooled statistics, consistent with GRADE guidance for narrative reviews. The GRADE informative statement templates proposed by Santesso et al. were used to frame conclusions at each certainty level [26].

### 2.7. Data Synthesis

Due to substantial clinical and methodological heterogeneity across included studies, quantitative meta-analysis was not performed. Studies varied considerably with respect to clinical populations, intervention modalities (e.g., ambient lighting, filtered lenses, LED exposure), exposure durations and intensities, comparator conditions, study designs, and outcome measures. Additionally, several studies employed crossover or within-subject designs with incomplete reporting of effect estimates and overlapping participant samples were identified in two migraine studies.

Therefore, findings were synthesized narratively and classified according to the mode of green light delivery, which determines the primary mechanism of retinal interaction. Three categories were identified based on the mechanistic literature [12] and used to organize the narrative synthesis: ambient green light exposure, green-tinted or wavelength filtering lenses, and red-free (green) examination light. The distinction between active green-wavelength stimulation and passive spectral filtering is mechanistically important: preclinical evidence indicates that green light analgesia requires cone receptor activation and is abolished by inhibition of the retino-vLGN pathway, whereas the analgesic mechanism of spectral filtering lenses may operate through reduction in ipRGC-mediated nociceptive signaling rather than through the same enkephalinergic circuit [13].

## 3. Results

### 3.1. Study Selection

**Figure 1:**
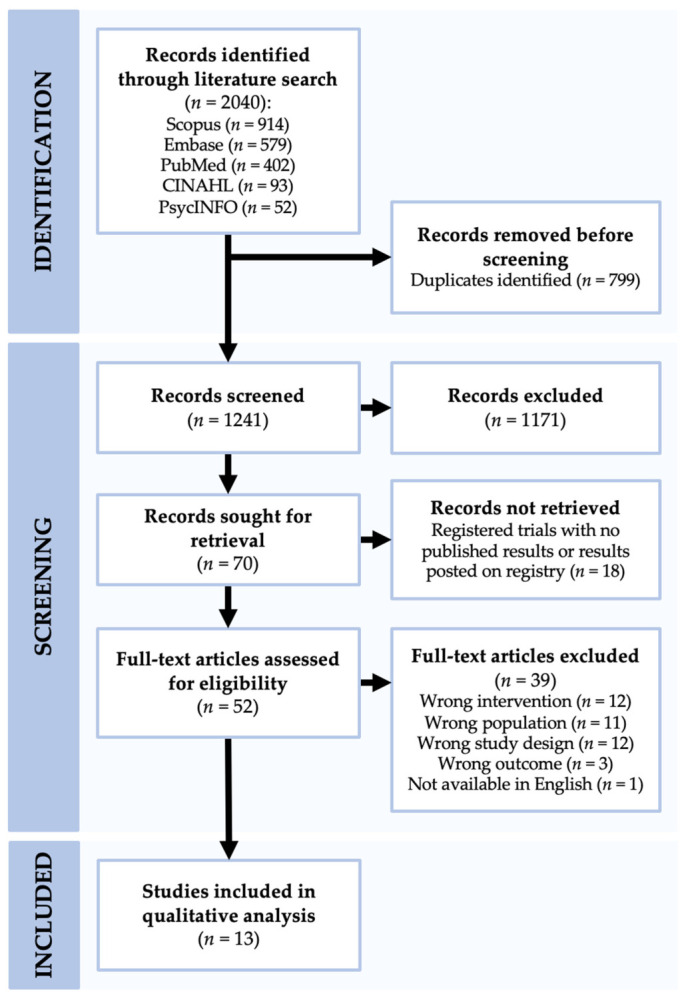
PRISMA flow diagram.

A total of 2040 citations were identified from database searches, of which 799 were duplicates. A total of 1241 citations were screened for inclusion using titles and abstracts, and 1171 were excluded for not meeting predefined eligibility criteria. Of the remaining 70 records sought for full-text retrieval, 18 could not be retrieved (ongoing or unpublished studies). The resulting 52 records were screened in full text, 39 were excluded for various reasons [27,28,29,30,31,32,33,34,35,36,37,38,39,40,41,42,43,44,45,46,47,48,49,50,51,52,53,54,55,56,57,58,59,60,61,62,63], and 13 studies were included. The study selection process is outlined in Figure 1.

### 3.2. Study Characteristics

Thirteen studies met the inclusion criteria and were included in this systematic review. Nine were randomized or quasi-randomized controlled trials (Table 1), and four were non-randomized studies of interventions (Table 2).

Studies were published between 2016 and 2025 and conducted across five countries: the United States (*n* = 7), Japan (*n* = 3), Turkey (*n* = 1), India (*n* = 1), and Pakistan (*n* = 1). The total number of participants across all 13 studies was approximately 668 total participants; however, this count overestimates the number of unique individuals because Noseda et al. 2016 [76] and Nir et al. 2018 [75] share overlapping participant data from the same research group (see Table 2, footnote †). After accounting for this overlap, the estimated number of unique participants is approximately 624.

#### 3.2.1. Randomized Studies (*n* = 9)

These studies addressed a range of clinical conditions: dental anxiety and procedural stress during intravenous sedation [64,71], migraine [65,66,70], dental anxiety and pain during first time third-molar extraction [68], behavioral cooperation and pain in children with autism spectrum disorder during dental prophylaxis [67], patient comfort during retinal examination [72], and fibromyalgia pain in patients on chronic opioid therapy [69]. Interventions included green-colored goggles (530–547 nm), green-tinted lenses, green-colored glasses (532 nm), green-light filtering eyeglasses, optical filter spectacles transmitting green wavelengths (500–570 nm), green light-exposed dental operatory, green (red-free) filter on binocular indirect ophthalmoscope, and lamps emitting green light (525 ± 10 nm). Exposure durations ranged from a brief exam [72] to a single 5-min session [65] to daily use for 4 weeks [66]. Comparators included translucent or clear-lens glasses, no lenses, blue-tinted lenses, red-colored goggles, blue-filtering eyeglasses, white-light environments, sham Transcranial Direct Current Stimulation (tDCS), yellow filter for ophthalmoscopy, and low-filtering control spectacles. Study designs included parallel-group RCTs [68,69,70,72], crossover RCTs [67,71], and within-subject repeated measures designs with randomized condition order [64,65].

#### 3.2.2. Non-Randomized Studies (*n* = 4)

Four additional non-randomized studies expanded the evidence base for ambient/filtered green light. These addressed migraine headache frequency and quality of life [73,75,76], as well as fibromyalgia pain and quality of life [74]. Interventions included narrow-band green LED exposure (525 nm) and calibrated green light stimuli at controlled intensities. Exposure durations ranged from single brief laboratory sessions [75,76] to 10 weeks of daily 1–2 h home exposure [73,74]. Comparators included white light LED exposure (same duration/intensity) and other wavelength light stimuli (blue, amber, red). Study designs included one-way crossover trials [73,74] and within-subject psychophysical studies with fixed stimulus order [75,76].

#### 3.2.3. Outcome Measures

Outcome measures varied substantially across studies. Pain was the most assessed outcome, measured using VAS (0–10 or 0–100 mm), the 11-point NRS or NPS (0–10), Short-Form McGill Pain Questionnaire (SF MPQ), the Faces Pain Scale-Revised (FPS-R), and the revised FLACC scale. The Numeric Pain Scale (NPS) and Numerical Rating Scale (NRS) are functionally equivalent self-report instruments for pain intensity, both commonly employing an 11-point (0–10) scale; the terminology used in this review reflects the nomenclature reported in each original study. Psychosocial outcomes included anxiety (VAS, STAI-S, VAS-DAS, VARS, PROMIS-57 anxiety domain), physiological stress markers (salivary alpha-amylase, heart rate, blood pressure, heart rate variability, pupillometry, salivary cortisol), photophobia/light sensitivity (VAS, binary presence/absence, headache exacerbation ratings), headache frequency (headache days per month), quality of life (PROMIS-57 domains, headache impact, Multidimensional Pain Inventory, Migraine-Specific Quality of Life), behavioral cooperation (FBRS, VBRS), opioid consumption (oral morphine equivalents), patient comfort during examination (non-validated questionnaire), and patient preference/satisfaction.

#### 3.2.4. Excluded and Ongoing Studies

A total of 39 studies were excluded at the full-text screening stage. The most common reasons for exclusion were wrong intervention (*n* = 12), wrong population (*n* = 11), wrong study design (*n* = 12), wrong outcome (*n* = 3), and not available in English (*n* = 1). Full details of excluded studies [27,28,29,30,31,32,33,34,35,36,37,38,39,40,41,42,43,44,45,46,47,48,49,50,51,52,53,54,55,56,57,58,59,60,61,62,63] and reasons for exclusion are provided in Table S2. An additional 18 registered clinical trials were identified as ongoing, not yet started, or completed without a published report at the time of the search; these are listed in Table S3.

### 3.3. Risk of Bias

#### 3.3.1. Randomized Controlled Trials (RoB 2)

Risk of bias was assessed using the Cochrane Risk of Bias 2 (RoB 2) tool for the outcomes identified in the nine included RCTs. Domain-level and overall judgments are presented in Figure S1. No RCT achieved an overall low risk of bias rating for any outcome. For the critical outcome of pain, five studies were judged as overall high risk of bias (Mahmood et al. 2025 [66], Nelli et al. 2023 [69], Posternack et al. 2023 [70], Takemura et al. 2021 [71], and Sharma et al. 2022 [72]). Four were rated as having some concerns (Takemura et al. 2025 [64], Kamata et al. 2025 [65], Sawicki et al. 2024 [67], and Gürses et al. 2024 [68]).

Domain 2 (deviations from intended interventions) was a frequently identified source of bias, which was rated as some concern or high risk in all studies for all outcomes. For Domain 2, this largely reflects the inherent difficulty of blinding participants and personnel to visible light-based interventions, as participants are generally able to perceive the color of goggles, lenses, or ambient lighting, and treating clinicians are often aware of the assigned condition. For Domain 4, this is primarily related to the use of subjective self-reported outcomes (e.g., VAS pain, VAS anxiety, Numeric Pain Scale, Migraine-Specific Quality of Life) assessed by participants who were not fully blinded to their allocation. Domain 3 (missing outcome data) was generally well-handled, although two studies (Mahmood et al. 2025 [66] and Nelli et al. 2023 [69]) were rated as high risk due to notable differential attrition. Domain 1 (randomization process) was high risk for outcomes assessed in two studies (Gürses et al. 2024 [68] and Sharma et al. 2022 [72]) due to use of a coin toss method without audit or allocation concealment, resulting in unequal groups and potentially imbalances in the baseline primary outcome values.

Reclassification of Takemura et al. 2025 [64] from an observational design to a randomized crossover RCT for the purposes of this review was due to the study incorporating a three-arm intervention and randomization of the order of conditions. This reclassification did not materially alter the overall pattern of risk of bias findings across included studies.

#### 3.3.2. Non-Randomized Studies (ROBINS-I)

Among the four non-randomized studies, there were three outcomes (pain, photophobia/light sensitivity, and quality of life) assessed with ROBINS-I. For the primary outcome of pain, all studies were judged as having overall serious risk of bias (Martin et al. 2021a [73]; Martin et al. 2021b [74]; Nir et al. 2018 [75]; Noseda et al. 2016 [76]). Domain-level and overall judgments are presented in Figure S2.

The most common source of potential bias across the outcomes in the non-randomized studies was confounding (Domain 1), rated as serious for all studies across all outcomes except for photosensitivity, which was judged as a moderate risk in one study (Noseda et al. 2016 [76]). The two one-way crossover trials by Martin et al. [73,74] employed a fixed sequence in which all participants received white light exposure prior to green light exposure, which may limit the ability to distinguish the intervention effect from time-related factors, natural disease fluctuation, regression to the mean, and the evolving therapeutic relationship. Similarly, Noseda et al. 2016 [76] and Nir et al. 2018 [75] used a fixed (non-randomized) order of light color stimuli, which may make it difficult to separate order effects from wavelength effects in the pain outcome measures, but was unlikely to be impactful for more objective assessments used for photophobia or light sensitivity outcomes. Additionally, these studies did not account for potential confounders such as medication use, comorbidities, or sex imbalance.

Measurement of outcomes (Domain 6) was the second most frequently identified source of potential bias, judged as serious in all four studies assessing pain outcomes. All primary outcomes in the non-randomized studies were self-reported by participants who were aware of the light color condition. Selection of reported results (Domain 7) was rated as moderate, in which multiple comparisons were performed without correction or where flexible analytic strategies were used.

Inter-reviewer risk-of-bias ratings had complete concordance across all domain-level and overall risk-of-bias judgements for every study, and no ratings were modified during the verification process.

### 3.4. Ambient Green Light: Results of Individual Studies

#### 3.4.1. Ambient Green Light: Pain Outcomes

Six studies directly assessed pain outcomes in the context of ambient green light. Results were inconsistent across studies, with the direction and significance of findings varying by study design, comparator, population, and methodological rigor. For non-randomized studies, the direction of effect favored green light, while randomized studies had mixed results.

Mahmood et al. 2025 [66] conducted a three-arm parallel-group RCT (*n* = 69 completers of 89 randomized) comparing anodal tDCS, sham tDCS, and green light therapy (525 ± 10 nm, 1 h/day, 5 days/week for 4 weeks) in migraine patients. In between-group analysis, significant reductions in pain intensity (Numeric Pain Scale) were observed at 2 and 4 weeks (*p* < 0.001) with effect sizes of 0.730 and 0.706, respectively. Significant reductions in pain interference (Multidimensional Pain Inventory) were observed at 2 weeks (*p* < 0.001; effect size of 0.35) and 4 weeks (*p* < 0.001; effect size of 0.52). Significant reductions in pain severity (Multidimensional Pain Inventory) were observed at 2 weeks (*p* < 0.001; effect size of 0.41) and 4 weeks (*p* < 0.001; effect size of 0.61). Within the Multidimensional Pain Inventory, multiple other areas demonstrated significant change, including affective distress, distracting response, household chores, outdoor work, activities away from home, and social activities, all reaching *p* < 0.001 at 4 weeks. A significant reduction in total migraine attacks per episode (structured headache diary) after exposure to the intervention, with the paper emphasizing this result for the tDCS group. Within-group analysis demonstrated all three groups had effects on the reduction in pain intensity via the Numeric Pain Scale (*p* < 0.001). For the green light group specifically, the MPI subscales significantly improved, indicating a decrease in the impact of pain and an increase in participation in everyday activities.

Sawicki et al. 2024 [67] evaluated a green-light dental operatory versus standard white-light operatory in children with ASD (*n* = 12 completers) in a crossover design. No significant differences were found for pain outcomes (FPS-R *p* = 0.50, r-FLACC *p* = 0.17).

Martin et al. 2021a [73] evaluated green LED exposure (525 nm, 1–2 h daily for 10 weeks) versus white LED exposure (10 weeks) in a one-way crossover design in migraine patients. The study reported significant reductions in headache days per month during the green light phase in both episodic migraine (*p* < 0.05) and chronic migraine (*p* < 0.001) subgroups. WLED group demonstrated significant reductions in headache days per month only when all patients (episodic and chronic migraine) were combined (*p* < 0.05). Post-hoc analysis of responder rates found that 86% (6/7) of episodic migraine patients and 63% (12/19) of chronic migraine patients achieved greater than 50% reduction in headache days per month during the GLED phase.

Martin et al. 2021b [74] used an identical one-way crossover design in fibromyalgia patients, reporting significant reductions in NRS pain intensity (*p* < 0.0001) during the green light phase (8.4 ± 0.3 to 4.9 ± 0.4 for GLED; 8.7 ± 0.2 to 8.1 ± 0.4 for WLED). Short-Form McGill Pain Questionnaire (SF MPQ) also was reported to demonstrate a statistically significant change from baseline, with WLED improving 6/15 descriptors and GLED improving 12/15 descriptors (*p* < 0.05).

Nir et al. 2018 [75] and Noseda et al. 2016 [76] conducted within-subject psychophysical studies examining color-selective photophobia in migraine patients. Both studies reported that green light exacerbated headache in a significantly smaller proportion of patients than other wavelengths (blue, amber, red, white) across a range of intensities. Noseda et al. 2016 [76] additionally reported that green light was the only color that did not produce a significant change from baseline pain ratings when data across all intensities were pooled (*p* = 0.7), whereas white, blue, amber, and red each produced significant increases (*p* < 0.0001). Green light also reduced pain intensity in 20% of patients, and at low intensities (1 and 5 cd·m^−2^), green was the only color to induce a decrease in pain ratings, with a reduction of 15%. Furthermore, green light induced less throbbing, muscle tenderness, and headache spread compared to blue, amber, and red.

#### 3.4.2. Ambient Green Light: Psychosocial Outcomes

Four studies assessed ambient green light with anxiety, mood, or quality of life outcomes. Sawicki et al. 2024 [67] assessed anxiety and reported no significant differences between the control and treatment conditions (*p* = 0.31) in the VARS assessment. Martin et al. 2021a [73] reported improvements in several quality-of-life measures during the green light phase in migraine patients. Quality of life assessed using the EQ-5D-5L demonstrated significant improvement following both white LED and green LED exposure in episodic migraine, chronic migraine, and combined migraine groups, though the improvement was more pronounced after green LED exposure. Notably, patients did not report significant improvement in self-perceived health following white LED exposure but did report significant improvement following green LED exposure across all migraine subgroups. WLED exposure did not improve HIT-6 scores in any migraine subgroup (EM, CM, or combined). GLED exposure produced a significant reduction in HIT-6 scores across all migraine subgroups, indicating improved functional capacity.

Martin et al. 2021b [74] similarly reported improvements in quality of life during the green light phase in fibromyalgia patients. While patients reported a small but statistically significant improvement in the FIQ score after exposure to WLED (*p* = 0.012), GLED exposure was shown to produce a significantly greater improvement in the quality-of-life measure (*p* < 0.0001). Mahmood et al. 2025 [66] reported significant improvements in Migraine-Specific Quality of Life in the green light group compared to sham tDCS (*p* < 0.001) with effect sizes of 0.59 at 2 weeks and 0.70 at 4 weeks.

#### 3.4.3. Ambient Green Light: Physiological Stress Markers

Only one study assessed ambient green light with physiological stress markers. Sawicki et al. 2024 [67] measured heart rate, salivary cortisol, and salivary alpha-amylase (AMY1) in children with autism spectrum disorder (*n* = 12) during dental prophylaxis performed under green-light versus white-light operatory conditions in a counterbalanced crossover design. Heart rate was similar at the start and end of each visit in both conditions and was not modified by treatment (*p* = 0.41). Similarly, there were no significant differences in salivary cortisol concentration (*p* = 0.67) or salivary AMY1 (*p* = 0.19) concentration between the green-light and white-light conditions.

#### 3.4.4. Ambient Green Light: Photophobia/Light Sensitivity

Noseda et al. 2016 [76] assessed color-selective migraine photophobia during acute untreated migraine attacks in 41 patients with normal eyesight who self-reported photophobia. Patients were exposed to white, blue, green, amber, and red lights at five calibrated intensities. At the highest intensity, nearly 80% of patients reported headache exacerbation with white, blue, amber, and red lights, whereas green light affected approximately half that proportion.

Nir et al. 2018 [75] extended these findings by comparing the same light exposure paradigm across ictal migraineurs (*n* = 44, with data reused from Noseda et al. [76]), interictal migraineurs (*n* = 59), and healthy controls (*n* = 17). During the ictal phase, the proportion of patients reporting headache exacerbation increased with light intensity across all colors (*p* < 0.01); at the highest intensity (100 cd·m^−2^), nearly 80% of patients demonstrated headache intensification with all colors except green, which affected approximately half that proportion. During the interictal phase, a similar intensity-dependent pattern was observed (*p* < 0.01). At the highest intensity, white, blue, amber, and red triggered new headaches in 17%, 19%, 16%, and 19% of patients, respectively, whereas green triggered headache in only 3%. When data across all intensities were pooled, the proportions of patients with new-onset headache were comparable across white, blue, amber, and red (*p* = 0.336), but a significantly smaller proportion reported new-onset headache in response to green compared to all other colors (*p* = 0.002). Among healthy controls, no headaches were reported at any intensity for white, blue, green, or amber; the only case of headache perception was in response to the highest intensity of red light.

#### 3.4.5. Ambient Green Light: Medication Use and Opioid Consumption

Three studies specifically stated medication dependency/use or opioid consumption as distinct outcomes in ambient green light interventions. Mahmood et al. 2025 [66] reported medication dependency was reduced across all groups, with the active tDCS group showing the most pronounced effect (95.7% reporting no medication use at 4 weeks). The green light group similarly showed reduced medication use, though specific proportions were not separately reported for this group. Martin et al. 2021a [73] stated pain medication use as an outcome; however, no conclusions regarding the reduction in pain medication in either the control or GLED treatment group were possible due to subjects reporting varied pain medication use for reasons unrelated to migraine. Martin et al. 2021b [74] similarly identified pain medication reduction as an outcome in fibromyalgia patients but reported that assessing medication reduction was challenging because patients were on pharmacologically different classes of medication (e.g., nonsteroidal anti-inflammatory drugs, muscle relaxants, opioids). Formal averaging and comparison of pain medication reduction was therefore not possible; however, 11 of 21 patients self-reported that they had reduced their habitual pain medications, including opioids, while being exposed to green light.

#### 3.4.6. Ambient Green Light: Behavioral Cooperation and Treatment Satisfaction

Sawicki et al. 2024 [67] assessed behavioral cooperation, using the FBRS and VBRS in children with ASD during dental procedures. No significant differences were found between the green-light and white-light conditions in either FBRS (*p* = 0.18) or VBRS (*p* = 0.06). While not significant, children who received a dental prophylaxis in a green light-exposed dental operatory did show a trend toward reduced uncooperative behavior when assessed by the VBRS. These null pain and behavioral cooperation outcomes are consistent with the null physiological findings reported in the same study.

### 3.5. Green-Tinted/Filtering Lenses: Results of Individual Studies

#### 3.5.1. Green-Tinted/Filtering Lenses: Pain Outcomes

Five studies directly assessed pain outcomes in the context of filtered green light. Results were inconsistent across studies, with the direction and significance of findings varying by study design, comparator, population, and methodological rigor. For non-randomized studies, the direction of effect favored green light, while randomized studies had mixed results.

Takemura et al. 2025 [64] evaluated green, blue, red, and translucent goggles in dental patients with phobia (*n* = 20) in a within-subject design. At the time just before PIC (anticipated peak stress time point), VAS pain scores were significantly lower in the green condition compared to both red (*p* = 0.041) and translucent (*p* = 0.046) conditions, and significantly lower in the blue condition compared to red (*p* = 0.014) and translucent (*p* = 0.014). No significant difference was observed between the green and blue conditions.

Gürses et al. 2024 [68], the largest study in this subgroup (*n* = 128), evaluated green-lens glasses versus clear-lens glasses during third-molar extraction in a parallel-group RCT. Intraoperative pain (VAS) was significantly lower in the green-lens group (*p* = 0.045), though the effect size was small (Cohen d = 0.36).

Nelli et al. 2023 [69] evaluated green-light filtering eyeglasses versus clear-light filtering eyeglasses and blue-light filtering eyeglasses in fibromyalgia patients on chronic opioids (*n* = 34 completers) in a three-arm pilot study. For the secondary outcome of pain score (NRS) change, there was no significant difference across groups (*p* = 0.62). Linear regression estimated a mean difference (95% confidence interval (CI)) of −0.10 (−1.25, 1.06) for green versus clear (*p* = 0.86) and 0.22 (−1.0, 1.4) for blue versus clear (*p* = 0.71). The rate of pain score decline was numerically highest in the green group (67%) compared to blue (50%) and clear (45%), but this difference was not statistically significant (*p* = 0.56). Logistic regression similarly showed no significant difference in pain score decline between green and clear (*p* = 0.31) or between blue and clear (*p* = 0.84). Thus, neither green-light nor blue-light filtering eyeglasses demonstrated a statistically significant effect on pain scores compared to clear-light filtering eyeglasses.

Posternack et al. 2023 [70] conducted the only double-blind, multi-center, placebo-controlled trial (*n* = 78), evaluating ipRGC-filtering spectacles that selectively transmit green wavelengths (500–570 nm) versus low-filtering control spectacles in episodic migraine. The pre-specified primary efficacy endpoint, mean pain score reduction at 2 h for the first severe/very severe headache, was not met (*p* = 0.65). The pre-specified secondary endpoint at 4 h was also not significant (*p* = 0.87). However, post-hoc analyses using a mixed-effects model applied to all headaches with baseline pain ≥2 (modified ITT and per-protocol populations) found statistically significant pain reductions favoring the ipRGC lenses at 2 h (*p* = 0.021) and 4 h (*p* = 0.019).

Takemura et al. 2021 [71] evaluated green-colored glasses versus clear glasses during peripheral intravenous cannulation in dental patients requiring sedation (*n* = 24) in an open-label crossover design. VAS pain was significantly lower in the green condition (*p* = 0.011).

#### 3.5.2. Green-Tinted/Filtering Lenses: Psychosocial Outcomes

Three studies assessed green-tinted/filtering lenses with anxiety, mood, or quality of life outcomes. Gürses et al. 2024 [68] reported the most robust findings. The primary outcome was anxiety score change, and the green-lens group demonstrated a significant reduction in dental anxiety compared to the clear-lens group on both the VAS-based dental anxiety score (DAS) (–10.39 [95% CI, –12.99 to –7.79] vs. 3.69 [95% CI, 1.65 to 5.73]; Welch *t* test, 8.51; *p* < 0.001; Cohen d, 1.50) and the STAI-S (–8.16 [95% CI, –10.11 to –6.09] vs. 1.27 [95% CI, –0.78 to 3.31]; *p* < 0.001), with the clear-lens group showing an increase in anxiety over the preoperative waiting period while the green-lens group showed a decrease. There was good intraclass correlation between the two anxiety measures.

Nelli et al. 2023 [69] found the change in PROMIS anxiety domain score in green-light vs. white-light filtering eyeglasses was not significant (95% CI [−9.8, 1.4]; *p* = 0.138). However, they reported statistically significantly higher odds of PROMIS anxiety score reduction in the green-light filtering eyeglasses group when compared to the clear-light filtering eyeglasses group (OR [95% CI] 6.00 [1.02, 35.37]; *p* = 0.048), though both findings emerged from one of seven PROMIS domains tested without correction for multiple comparisons. Takemura et al. 2021 [71] found a non-significant trend toward lower VAS anxiety in the green condition (*p* = 0.109).

#### 3.5.3. Green-Tinted/Filtering Lenses: Physiological Stress Markers

Four studies assessed physiological stress markers in investigations of green-tinted/filtering lenses. Takemura et al. 2025 [64] found that the green condition produced significantly lower sAA activity than the translucent condition at the time point just prior to PIC (*p* = 0.009), while the blue condition exhibited a trend toward lower values compared to translucent that did not reach statistical significance. No significant differences in sAA were found among the green, blue, and red conditions, and no significant differences in heart rate were observed across the four conditions. In 2021, Takemura et al. 2021 [71] found that salivary alpha-amylase (sAA) increased significantly during peripheral intravenous cannulation in the clear-glasses condition (*p* < 0.001) but not in the green-glasses condition (*p* = 0.362), with a significant between-group difference (*p* = 0.025). However, no significant between-group differences were found for hemodynamic parameters (SBP, DBP, MBP, HR).

Gürses et al. 2024 [68] found a significant reduction in heart rate in the green-lens group compared to the clear-lens group (–6.17 [95% CI, –8.22 to –4.13] vs. –1.95 [95% CI, –4.79 to 0.88]; *t* test, 2.41; *p* = 0.017; Cohen d, 0.43), providing partial objective corroboration of the subjective anxiety findings, but no significant differences in blood pressure (systolic tension mean, *p* = 0.093; diastolic pressure mean, *p* = 0.427) or oxygen saturation (*p* = 0.597). Kamata et al. 2025 [65] measured heart rate variability (HRV) using frequency-domain analysis, with normalized high-frequency power (HFnu) as a parasympathetic index and normalized low-frequency power (LFnu) as a sympathetic index. Under the no-lens and blue-lens conditions, migraine patients exhibited decreased HFnu and significantly increased LFnu compared to healthy controls, suggesting sympathetic predominance. Under the green-tinted lens condition, these between-group differences in HFnu and LFnu were no longer statistically significant.

#### 3.5.4. Green-Tinted/Filtering Lenses: Photophobia/Light Sensitivity

Two studies assessed photophobia/light sensitivity in investigations of green-tinted/filtering lenses. Posternack et al. 2023 [70] found no significant difference in light sensitivity on pre-specified analyses assessed at 2 h (*p* = 0.19) and 4 h (*p* = 1.0) in subjects (*n* = 54) experiencing severe headaches. A post hoc mixed-effects model re-analyzed all headaches with baseline pain scores between 2 and 10 and found a significant effect at 2 h (*p* = 0.028) but not at 4 h (*p* = 0.46).

Kamata et al. 2025 [65] assessed VAS photophobia and reported scores remained significantly higher in migraine patients than controls under all lens conditions, including green, indicating that the green lenses did not normalize subjective light sensitivity despite the apparent changes in autonomic indices. Regarding pupillary constriction, no significant differences were observed between the migraine and control groups across any of the lens conditions. However, within-group comparisons showed that green lenses significantly reduced the pupil constriction rate compared to both the no-lens and blue-lens conditions in both migraine patients (no lens vs. green, *p* = 0.02; blue vs. green, *p* = 0.03) and controls (no lens vs. green, *p* < 0.001; blue vs. green, *p* = 0.01), with no significant difference between the no-lens and blue-lens conditions in either group.

#### 3.5.5. Green-Tinted/Filtering Lenses: Medication Use and Opioid Consumption

Two studies specifically stated medication dependency/use or opioid consumption as distinct outcomes in studies of green-tinted/filtering lenses.

Nelli et al. 2023 [69] was the only study to assess opioid consumption in the context of ambient green light therapy via green-light filtering eyeglasses versus clear or blue-light filtering eyeglasses. The primary efficacy outcome (≥10% decline in oral morphine equivalents OME at 2 weeks) did not reach statistical significance. However, logistic regression suggested a trend favoring the green-light filtering eyeglasses group, with an estimated 5.5-fold higher odds of achieving the ≥10% OME reduction compared to the clear group (95% confidence interval [CI] [0.66, 119]; *p* = 0.159).

Posternack et al. 2023 [70] reported that among headaches with a baseline pain score ≥2 for which medication use was recorded, 22% of headaches in the ipRGC-lens group required abortive migraine medication compared to 35% in the control-lens group. However, medication use was not a pre-specified outcome and was instead used as a covariate in the post-hoc mixed-effects model analyses; no formal statistical comparison of medication use between groups was reported.

#### 3.5.6. Green-Tinted/Filtering Lenses: Behavioral Cooperation and Treatment Satisfaction

Takemura et al. 2025 [64] found median VAS scores for treatment satisfaction were not statistically significantly different among the four conditions.

### 3.6. Red-Free (Green) Examination Light: Results of Individual Studies

#### 3.6.1. Red-Free (Green) Examination Light: Pain Outcomes

Sharma et al. 2022 [72] compared green (red-free) versus yellow light using a binocular indirect ophthalmoscope for retinal examination in 100 patients. Patients reported significantly greater comfort (*p* < 0.001) and less pain (*p* = 0.001) with green light, and 72% preferred green over yellow light. Clinician-rated patient cooperation was also significantly higher (*p* < 0.001) with green light (85.5%) in comparison to yellow light (24.4%).

#### 3.6.2. Red-Free (Green) Examination Light: Behavioral Cooperation and Treatment Satisfaction

Sharma et al. 2022 [72] reported higher clinician-rated patient cooperation during retinal examination with green (red-free) light compared to yellow light; however, this was assessed using a non-validated questionnaire in an unblinded design, limiting confidence in the finding.

### 3.7. Certainty of Evidence

The certainty of evidence was assessed using the GRADE framework for each outcome, stratified by comparison type: sham/white light (Comparison A) or a different non-white wavelength (Comparison B). Detailed GRADE Evidence Profile tables with domain-level justifications for each downgrading decision are provided in Tables S2 and S3 (Supplementary Materials). Key findings from the certainty assessment are summarized below.

Across both comparisons, the risk of bias domain was rated very serious (−2) for most outcomes, with physiological stress markers and opioid consumption being judged as serious (−1) due to the more objective nature of the assessments used. This rating reflects the pervasive inadequacy of blinding: almost all primary outcomes were self-reported by unblinded participants using VAS, NRS, or validated questionnaires, and RoB 2 Domain 2 (deviations from intended interventions) was rated as some concerns or high risk in every contributing RCT for all outcomes. Half of contributing RCTs received an overall high risk of bias judgment, and all non-randomized studies were rated as serious or moderate overall risk by ROBINS-I. Publication bias was not downgraded for any outcome other than pain due to the lack of sufficient studies contributing to each outcome. However, the absence of prospective trial registration in the majority of studies, small-study predominance, overlapping authorship, and absence of clearly negative published studies are noted as limitations. The potential participant overlap between Noseda et al. 2016 [76] and Nir et al. 2018 [75] was considered when assessing inconsistency and imprecision for outcomes to which both studies contributed; their concordant findings were not treated as independent replication.

The inconsistency rating for pain in Comparison A warrants specific comment. The only double-blind, placebo-controlled trial (Posternack et al. 2023 [70]) failed both its pre-specified primary and secondary pain endpoints, while statistically significant pain reductions were reported exclusively by smaller, open-label studies or emerged from post-hoc analyses of the otherwise negative double-blind trial. This pattern, in which the direction of effect varies systematically with methodological rigor rather than randomly across studies, was judged as a serious inconsistency because it raises the possibility that the positive findings reflect expectation or placebo effects rather than a true treatment effect.

For quality of life in Comparison A, inconsistency was not downgraded despite the methodological limitations of contributing studies, because all three showed effects in the same direction. However, the Martin et al. [73,74] fixed-sequence crossover designs completely confound the intervention effect with time, and Mahmood et al. 2025 [66] had significant baseline imbalances and 22% attrition, so the consistent direction should be interpreted cautiously.

For the ambient/filtered green light interventions, the certainty of evidence was rated very low (⊕**◯◯◯**) for all outcomes across both comparisons. The primary drivers of downgrading were very serious risk of bias (reflecting inadequate blinding of subjective outcomes across most studies) and serious imprecision (reflecting predominantly small, underpowered studies).

The Summary of Findings tables for Comparison A and Comparison B are presented in Table 3 and Table 4. GRADE Evidence Profile for Comparison A and Comparison B can be found in Tables S4 and S5.

### 3.8. Synthesis Without Meta-Analysis

**Figure 2:**
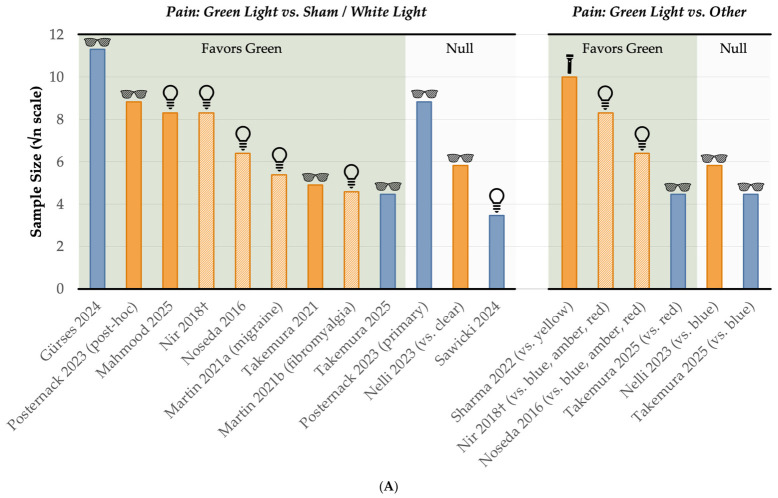

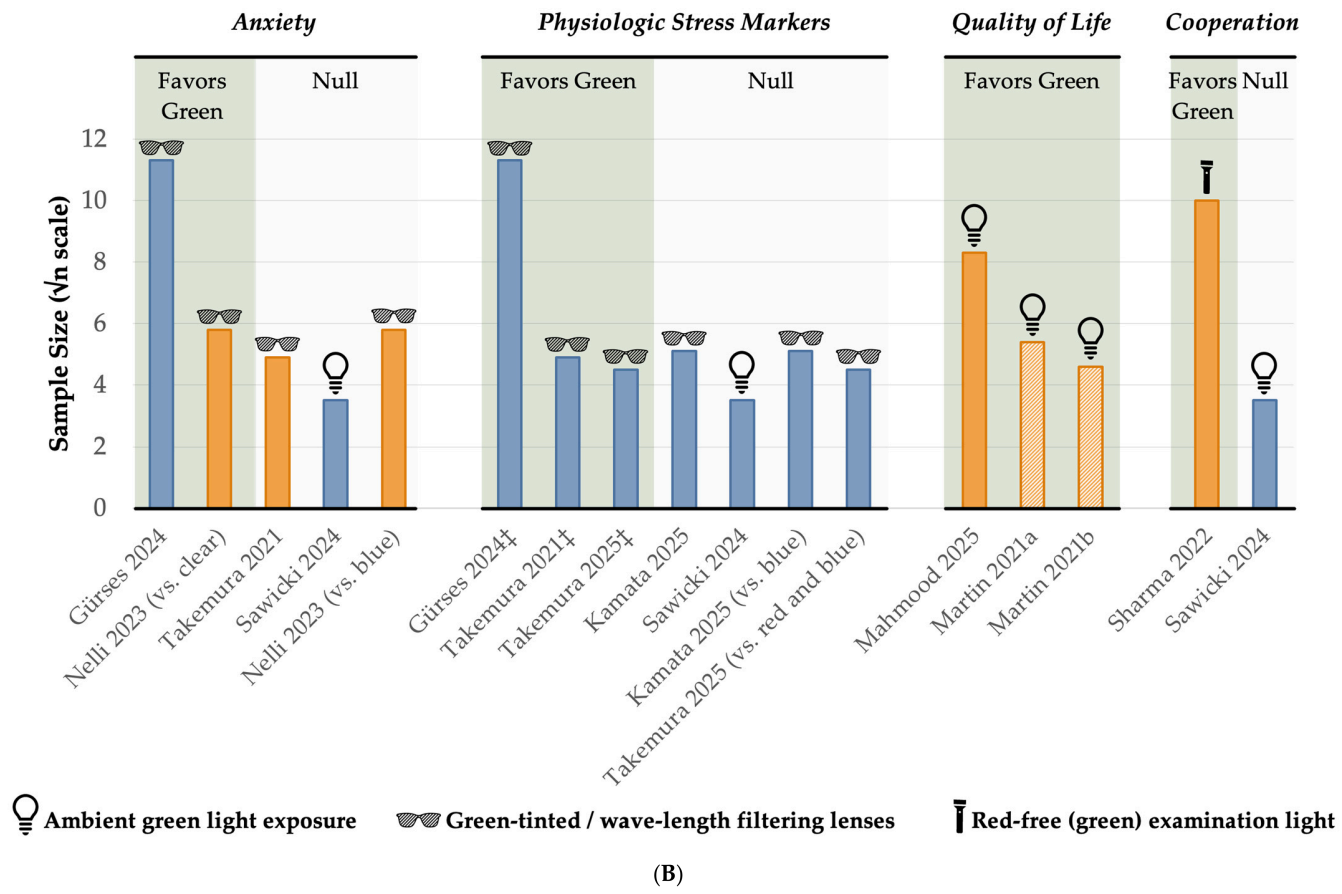
Modified harvest plots displaying the direction of effect, sample size, and risk of bias for (**A**) pain outcomes and (**B**) psychosocial outcomes across all 13 included studies. Each bar represents one study or one distinct analysis within a study (e.g., Posternack et al. 2023 [70] is represented separately for its pre-specified primary endpoint and its post-hoc analysis; studies contributing to multiple comparisons appear in both the green vs. sham/no treatment and green vs. other wavelengths facets). Bar height corresponds to sample size on a square-root scale for visual clarity. † = Studies with overlapping participant data indicating non-independence. ‡ = Mixed result: at least one physiological stress marker was statistically significant while others were null. Bar color indicates the overall risk of bias judgment: blue = some concerns (RoB 2) or moderate risk (ROBINS-I); orange = high risk (RoB 2) or serious risk (ROBINS-I). Bar pattern (solid = RCT; diagonal lines = non-randomized study) distinguishes study design. Position along the horizontal axis indicates the direction and statistical significance of the effect: leftward positions indicate results favoring green light; rightward indicates null or no significant difference. No study favored the comparator [64,65,66,67,68,69,70,71,72,73,74,75,76].

Because the heterogeneity of interventions, populations, comparators, and outcome measures precluded meta-analytic pooling, the results of individual studies were synthesized narratively. Modified harvest plots (Figure 2) were used to provide a visual overview of the direction of effect, sample size, and risk of bias across studies for pain (Figure 2A) and psychosocial outcomes (Figure 2B), following the Synthesis Without Meta-analysis (SWiM) reporting guideline.

#### 3.8.1. Pain Outcomes (Figure 2A)

For the green light vs. sham/white light comparison, the majority of studies reported pain reductions favoring green light. However, the positive findings were predominantly derived from open-label or unblinded designs. Among the studies reporting statistically significant pain reductions favoring green light, Gürses et al. 2024 [68] (*n* = 128, some concerns) was the largest, though the effect size was small (Cohen d = 0.36) for green-colored glasses and intraoperative pain. The remaining positive findings came from smaller studies: Mahmood et al. 2025 [66] (*n* = 69, high risk), Takemura et al. 2021 [71] (*n* = 24, high risk), and Takemura et al. 2025 [64] (*n* = 20, some concerns). Among the non-randomized studies, Nir et al. 2018 [75] (*n* = 69 †, serious risk) and Noseda et al. 2016 [76] (*n* = 41, serious risk) found green light was significantly less exacerbating than white light during both ictal and interictal migraine phases, though these represent perceptual findings with shared participant data and fixed stimulus order. Martin et al. 2021a [73] (*n* = 29, serious risk) and Martin et al. 2021b [74] (*n* = 21, serious risk) reported significant pain reductions during the green light phase, though the fixed-sequence designs preclude separation of the intervention effect from temporal confounding. Posternack et al. 2023 [70] (*n* = 78, some concerns but double-blind with pre-specified null result) failed both its pre-specified primary and secondary pain endpoints; statistically significant findings emerged only from post-hoc analyses. Nelli et al. 2023 [69] (*n* = 34, high risk) did not find a significant difference in pain score change or decline in green-light filtering eyeglasses over clear-light filtering eyeglasses. Sawicki et al. 2024 [67] (*n* = 12, some concerns) found no significant pain differences in children with ASD.

In the green light vs. other wavelengths comparison, Sharma et al. 2022 [72] (*n* = 100, high risk) reported greater comfort with green versus yellow light during retinal examination. Nir et al. 2018 [75] (*n* = 69 †, serious risk) and Noseda et al. 2016 [76] (*n* = 41, serious risk) found green light was the least exacerbating wavelength compared to blue, amber, and red. Takemura et al. 2025 [64] (*n* = 20, some concerns) found green goggles produced significantly lower pain than red (*p* = 0.041) but not blue goggles, and is therefore represented in both the “Favors Green” (vs. red) and “Null” (vs. blue) categories. Nelli et al. 2023 [69] (*n* = 34, high risk) did not find a significant difference in pain score change or decline in green-light versus blue-light filtering eyeglasses.

#### 3.8.2. Psychosocial Outcomes (Figure 2B)

For anxiety, the largest study, Gürses et al. 2024 [68] (*n* = 128), reported significant anxiety reduction on both DAS and STAI-S, with partial objective corroboration from heart rate reduction, but this was an open-label trial with subjective primary outcomes. Nelli et al. 2023 [69] (*n* = 34) reported a non-significant mean change in PROMIS Anxiety domain scores, though a secondary responder analysis found significantly higher odds of anxiety score reduction with green-light glasses vs. clear. Both results emerged from one of seven domains tested without multiplicity correction, and the odds ratio confidence interval barely excludes the null. Takemura et al. 2021 [71] (*n* = 24) found a non-significant trend toward lower VAS anxiety.

Physiological stress markers showed mixed results regardless of study size. Gürses et al. 2024 [68] (*n* = 128) found significant heart rate reduction favoring green light but no blood pressure differences. Takemura et al. 2021 [71] (*n* = 24) found significant sAA attenuation with green glasses but no hemodynamic differences. Takemura et al. 2025 [64] (*n* = 20) found significantly lower sAA with green versus translucent goggles but no significant differences among green, blue, and red conditions. Sawicki et al. 2024 [67] (*n* = 12) found no significant differences in heart rate, salivary cortisol, or salivary AMY1 between green and white light conditions. Kamata et al. 2025 [65] (*n* = 26) reported that green lenses appeared to normalize HRV indices in migraine patients, but no formal interaction test was performed and no significant differences were found in HRV between green and blue conditions when analyzed by condition. Takemura et al. 2025 [64] found no significant heart rate differences among green, red, and blue conditions.

For quality of life, all three contributing studies showed improvements favoring green light: Mahmood et al. 2025 [66] (*n* = 69, high risk), Martin et al. 2021a [73] (*n* = 29, serious risk), and Martin et al. 2021b [74] (*n* = 21, serious risk). However, the Martin studies used fixed-sequence designs, and Mahmood et al. had significant methodological limitations.

For behavioral cooperation, Sharma et al. 2022 [72] (*n* = 100, high risk) reported higher clinician-rated patient cooperation with green versus yellow light during retinal examination, though this was assessed using a non-validated questionnaire. Sawicki et al. 2024 [67] (*n* = 12, some concerns) found no significant differences between green and white light conditions.

#### 3.8.3. Other Outcomes

Photophobia/light sensitivity and medication use/opioid consumption were not included in the harvest plots as they represent distinct clinical outcomes rather than psychosocial measures; these outcomes are summarized in the GRADE Summary of Findings tables (Table 3 and Table 4).

For photophobia/light sensitivity, Posternack et al. 2023 [70] (*n* = 78) found no significant difference on pre-specified analyses, with positive findings limited to post-hoc analyses. Kamata et al. 2025 [65] (*n* = 26, migraine subgroup *n* = 10) found that VAS photophobia remained elevated under all lens conditions including green. Noseda et al. 2016 [76] (*n* = 41) and Nir et al. 2018 [75] (*n* = 69 †) found green light was the least exacerbating wavelength across intensities, but these laboratory-based perceptual findings with shared participant data and fixed stimulus order demonstrate a relative phenomenon rather than a therapeutic effect.

For medication use/opioid consumption, Mahmood et al. 2025 [66] (*n* = 69) reported reduced medication dependency across all groups; Martin et al. 2021a [73] (*n* = 29) could not draw conclusions due to varied medication use; Martin et al. 2021b [74] (*n* = 21) reported self-reported medication reductions in 11 of 21 patients without quantitative analysis, and Nelli et al. 2023 [69] (*n* = 34) found a non-significant trend favoring green-light filtering eyeglasses for opioid reduction.

## 4. Discussion

This systematic review evaluated the effects of ambient or filtered green light interventions on pain and psychosocial outcomes across 13 studies (9 randomized or quasi-randomized controlled trials and 4 non-randomized studies of interventions) encompassing approximately 668 participants (~624 unique individuals after accounting for participant overlap between Noseda et al. [76] and Nir et al. [75]). The evidence base is characterized by substantial heterogeneity in interventions, populations, comparators, and outcomes, which precluded meta-analytic pooling and necessitated a narrative synthesis with GRADE certainty assessment. Several considerations support this inclusive approach and provide coherence across the diverse included studies. First, all included studies involve modulation of the spectral composition of light reaching the retina, with green wavelengths playing a central role. However, presumed mechanisms differ by delivery method. Second, the biopsychosocial model of pain provides a unifying theoretical framework that links the diverse outcomes measured across studies [1]. Pain intensity, dental anxiety, procedural discomfort, and behavioral responses in neurodevelopmental conditions all reflect overlapping neurobiological processes. Third, the evidence base for green light therapy in humans remains small. Given the total number of published studies is limited, this systematic review can serve a critical function in mapping the breadth of populations and outcomes that have been investigated. The central finding is that the current evidence, while providing early signals of potential benefit, does not yet permit definitive conclusions regarding the efficacy of ambient or filtered green light for pain or psychosocial outcomes. The certainty of evidence was very low for all assessed outcomes across both comparisons (green light vs. sham/no treatment and green light vs. other wavelengths), reflecting the preliminary nature of the available studies rather than evidence of efficacy.

### 4.1. Interpretation of Findings

The 13 included studies addressed a broad range of clinical conditions, dental anxiety, migraine, fibromyalgia, autism spectrum disorder, and retinal examination discomfort, and used diverse delivery methods including colored goggles, tinted lenses, optical filter spectacles, ambient room lighting, and LED lamps. The majority of studies reported findings favoring green light, including the largest study with the lowest risk of bias (Gürses et al. 2024 [68]). However, nearly all positive results emerged from open-label or unblinded designs, which, while pragmatically appropriate for early-phase investigation, cannot isolate the specific contribution of green light from nonspecific factors such as participant expectation. Adequately blinded trials are therefore needed to confirm whether these promising findings reflect a true treatment effect.

The most methodologically robust study, Posternack et al. 2023 [70], a double-blind, multi-center, placebo-controlled, registered trial, failed both its pre-specified primary and secondary endpoints, with all statistically significant pain findings generated through post-hoc modifications to the study population, analytic method, and unit of analysis. This pattern suggests that blinding adequacy, rather than sample size alone, may be the critical methodological factor distinguishing positive from null results. Sharma et al. 2022 [72] provides pragmatically useful information, suggesting that patients may find green (red-free) light more comfortable than yellow light during indirect ophthalmoscopy. The positive pain findings from open-label studies cannot be confidently attributed to a specific analgesic effect of green light, as these designs do not permit differentiation between treatment-specific effects and nonspecific factors such as expectation or placebo responses.

The anxiety findings present a somewhat more consistent picture, with all three contributing studies showing effects in the direction favoring green light. However, the largest reported effect size (Gürses et al. 2024 [68], Cohen d = 1.50), substantially exceeding those of most pharmacological anxiolytics, was obtained in an open-label design that raises the possibility that nonspecific factors may have contributed to the observed difference, though specific anxiolytic effect cannot be excluded. The Nelli et al. 2023 [69] anxiety finding emerged from one of seven domains tested without multiplicity correction, and the mean change analysis was not significant, further limiting its interpretability.

Physiological stress markers, which are less susceptible to expectation bias than self-reported outcomes, showed inconsistent results. The significant salivary alpha-amylase findings in both Takemura et al. studies [64,71] are noteworthy as objective measures, but none of the included studies were adequately powered to detect physiological differences, and the absence of corresponding hemodynamic differences in both studies with the null physiological findings in Sawicki et al. 2024 [67] preclude firm conclusions about whether green light exerts a consistent autonomic effect. Additionally, the Takemura et al. 2025 [64] findings should be interpreted cautiously given the non-standardized dental procedures across visits. The Kamata et al. 2025 [65] interpretation that green-tinted lenses may normalize autonomic balance in migraine patients is methodologically limited, as the study compared separate between-group *p*-values under each condition rather than performing a formal group × condition interaction test; the disappearance of a significant difference under one condition does not constitute statistical evidence of a treatment effect.

The discordance between objective and subjective outcomes within and across studies highlights the difficulty of distinguishing specific physiological effects of green light from broadly defined responses in the current evidence base. Notably, preclinical studies have demonstrated antinociceptive and anxiolytic effects of green light through opioid-mediated, endocannabinoid, and neuromodulatory pathways in animal models [13,14,15,16], where placebo and expectation effects are not operative, supporting biological plausibility for a specific mechanism of action.

The inclusion of four non-randomized studies assessed with ROBINS-I reinforces rather than alters the conclusions drawn from the RCT evidence. The two one-way crossover trials by Martin et al. [73,74], evaluating green LED exposure for migraine and fibromyalgia, have been widely cited as supporting evidence but share a critical methodological limitation that has not been sufficiently emphasized in the literature. The fixed sequence design (white light first, green light second in all participants) confounds the observed improvements with time, natural disease fluctuation, regression to the mean, the cumulative effect of prolonged study participation, and the therapeutic relationship with the research team. Without a concurrent control group or randomized crossover, improvements cannot be confidently attributed to green light exposure rather than to these nonspecific temporal factors. Nevertheless, the magnitude and consistency of the reported improvements across trials motivated subsequent investigations and remain consistent with the biological plausibility established by preclinical evidence.

The psychophysical studies by Noseda et al. 2016 [76] (*n* = 41) and Nir et al. 2018 [75] provide the strongest mechanistic foundation, demonstrating that green light produces less exacerbation of headache pain than other wavelengths in migraine patients. These findings support the proposed mechanism involving cone-driven retinal pathways and thalamic modulation of pain processing. However, they may demonstrate a relative perceptual phenomenon rather than a therapeutic effect. The finding that green light is the least averse wavelength provides mechanistic support for the green light hypothesis but does not itself establish that prolonged green light exposure will produce clinically meaningful pain relief.

### 4.2. Delivery Method and Exposure Parameters as Potential Effect Modifiers

The included studies employed at least three distinct delivery methods that are unlikely to be equivalent interventions. Ambient/environmental light (LED lamps or room lighting) exposure requires sustained retinal exposure to green-wavelength photons at specific intensities and durations [18]. Wearable optical devices (goggles, tinted lenses, or filtering spectacles), by contrast, may exert their effects primarily by filtering out wavelengths (blue, amber, red) that activate stimulating green-specific analgesic circuits [70]. These delivery methods differ in the visual field coverage, spectral purity, and intensity of green light reaching the retina, which may influence the magnitude of any therapeutic effect.

The distinction between “adding green” and “subtracting non-green” is mechanistically significant and has implications for dose-response relationships, optimal exposure parameters, and generalizability across clinical conditions. Red-free ophthalmologic examination light represents a further departure where the green light serves a diagnostic purpose (enhancing retinal vascular contrast), exposure is measured in seconds to minutes rather than hours, and the clinical context differs fundamentally from therapeutic applications. The inclusion of such delivery in this review reflects the broad evidence-mapping objective but should not be interpreted as suggesting mechanistic equivalence with therapeutic green light protocols. These distinctions temper the conclusions that can be drawn from the pooled evidence. Readers should interpret the findings within each intervention category rather than generalizing across delivery methods.

Single-session wearable studies (Takemura et al. 2025 [64]; Gürses et al. 2024 [68]; Takemura et al. 2021 [71]) consistently reported pain reductions favoring green light, though all employed open-label designs, limiting the ability to distinguish specific from nonspecific effects. In contrast, the multi-session wearable studies (Nelli et al. 2023 [69]; Posternack et al. 2023 [70]) did not demonstrate significant effects on their pre-specified primary pain endpoints despite longer cumulative exposure durations. Nelli et al. 2023 [69] was designed as a feasibility pilot and was not powered for definitive efficacy conclusions; therefore, the findings should be interpreted as hypothesis-generating rather than confirmatory. It is also worth noting that the pre-specified primary endpoint in Posternack et al. 2023 [70], restricted to the first severe or very severe headache, may have selected for migraine attacks in which pain intensity was already too high for a non-pharmacological visual intervention to produce a detectable effect, whereas the post-hoc analysis evaluating all headaches with baseline pain ≥2 may better reflect the clinical scenario in which green light filtering could plausibly modulate pain at earlier stages of the headache course; this interpretation, while speculative, could inform endpoint selection in future trials.

Among the studies investigating ambient green light via LED or lamp (Mahmood et al. 2025 [66]; Sawicki et al. 2024 [67]; Martin et al. 2021a [73]; Martin et al. 2021b [74]), three of four reported positive pain findings with daily exposures of 1–2 h over 4–10 weeks. However, each had notable methodological limitations: the Martin et al. [73,74] studies used fixed-sequence designs in which the intervention effect cannot be readily separated from time-related factors, and Mahmood et al. [66] had significant baseline imbalances, high attrition, and no blinding. The null result in Sawicki et al. 2024 [67] involved children with autism spectrum disorder, in whom differences in sensory processing may influence the response to visual stimuli. The longer cumulative exposures in ambient green light studies compared to the single-session wearable studies could be consistent with a dose-response relationship, though this interpretation remains uncertain given the temporal confounding and higher risk of bias in the ambient/environmental light sources.

The calibrated light stimulus studies (Noseda et al. 2016 [76]; Nir et al. 2018 [75]) demonstrated that green light is the least exacerbating wavelength for migraine photophobia under laboratory conditions, establishing an important perceptual phenomenon that supports biological plausibility, though these findings do not directly address whether sustained exposure produces therapeutic benefit in clinical settings.

A clear dose-response relationship cannot be confidently established from the current evidence, as the studies with the largest cumulative exposures also had the most significant methodological limitations. Future studies would benefit from systematically varying exposure parameters within a single trial design to clarify optimal treatment conditions.

### 4.3. Cross-Cutting Methodological Concerns

Blinding. The inherent visibility of green light interventions presents a fundamental challenge to adequate blinding. Only Posternack et al. 2023 [70] achieved double-blinding, and even in this study, some degree of visual distinguishability may have partially compromised masking. In the remaining studies, the open-label or single-blind designs mean that the current evidence cannot reliably distinguish specific effects of green light from nonspecific factors on subjective outcomes. The RoB 2 assessment identified Domains 2 (deviations from intended interventions) and 4 (measurement of the outcome) as the most consistently affected (Figure S1), contributing to the very serious risk of bias downgrading in the GRADE assessment (Tables S2 and S3). Notably, the challenge of blinding light-based interventions is not unique to this field, and the development of credible sham controls should be a priority for future trials.

Sample size and statistical power. The median sample size across all 13 studies was 34 participants, and only one study enrolled more than 100 participants. Most studies did not report a priori power calculations, and those that did were often powered for primary outcomes other than pain. These sample sizes may have been insufficient to detect clinically meaningful but modest effect sizes, and future trials would benefit from formal power calculations based on the effect estimates observed in the current literature.

Heterogeneity of interventions. The term “green light therapy” encompasses a range of distinct interventions, from colored goggles worn for minutes during a dental procedure to optical filter spectacles worn for weeks during migraine attacks to ambient LED exposure for hours daily over several weeks. The mechanisms of action may differ depending on the spectral purity, intensity, and duration of retinal exposure. Grouping these interventions together, while necessary for a comprehensive review, may obscure meaningful differences in efficacy and mechanism that could be clarified through more targeted investigations.

Trial registration and reporting. Only 2 of 13 studies were prospectively registered. The absence of registration in the remaining studies prevents verification of pre-specified outcomes and analyses, underscoring the importance of prospective registration in future work.

Temporal confounding in crossover designs. The fixed-sequence crossover design used by Martin et al. [73,74] introduces confounding that is distinct from the blinding concerns, as the observed improvements during the green light phase cannot be separated from time-related factors. While these designs can generate useful preliminary data, randomized, counterbalanced designs with adequate washout periods would substantially strengthen causal inference.

Interpreting consistency of findings. Several recurring methodological limitations were identified, including unblinded subjective outcomes, temporal confounding, and small sample sizes. These factors generally operate in the same direction, potentially favoring the appearance of benefit for the intervention. When interpreting the consistency of positive findings across studies, it is important to consider that shared methodological vulnerabilities may contribute to concordant results. However, this does not preclude the possibility of a true treatment effect; rather, it underscores the need for adequately blinded, well-powered trials to confirm or refute the preliminary signals observed to date. This consideration is informed by the observation that the only double-blind RCT did not demonstrate significant pain effects on its pre-specified endpoints, though endpoint selection may have influenced this result.

Conflicts of interest. Financial relationships between investigators and industry were disclosed in several studies. While conflicts of interest are not a formal domain in either RoB 2 or ROBINS-I, transparency regarding funding sources and financial relationships is important context when interpreting study findings alongside other methodological considerations.

### 4.4. Comparison with the Existing Literature

The findings of this review are broadly consistent with the preclinical literature suggesting that green light exposure may modulate nociceptive processing through cone-dominated retinal pathways. Predominant preclinical models have demonstrated antinociceptive effects through mechanisms involving endogenous opioid and endocannabinoid systems, and these findings are important because placebo and expectation effects are unlikely to explain results obtained in animal models. Nonetheless, preclinical studies typically employ longer exposure durations, tightly controlled environmental lighting, and objective behavioral outcome measures, and the translation of these findings to the heterogeneous clinical studies reviewed here remains unestablished. The gap between the preclinical promise and the clinical evidence highlights the need for more rigorous translational research that carefully addresses the methodological limitations identified in this review, rather than suggesting that the therapeutic potential of green light is unlikely.

### 4.5. Implications for Future Research

The findings of this review suggest several opportunities for advancement of future research in this field:

Blinding strategies. The development of credible sham controls for green light interventions is essential. Potential approaches include spectrally matched control lenses that transmit similar total light intensity but different wavelength compositions, or the use of narrow-band green light versus broad-spectrum light of matched luminance.

Adequately powered trials. Based on the effect sizes observed in the current literature (where significant), sample sizes of 100–200 per arm would be needed to detect moderate effects (Cohen d = 0.3–0.5) with adequate power.

Standardized outcome measures. The use of IMMPACT-recommended pain outcome measures and standardized assessment time points would facilitate cross-study comparison and future meta-analysis.

Mechanistic clarity. Studies should clearly distinguish between ambient green light exposure (hypothesized to act through retinal-neural pathways) and other light-based interventions such as photobiomodulation (acting through direct tissue effects), as these represent fundamentally different interventions with different proposed mechanisms. Delivery methods should be explicitly compared and future research would benefit from investigating whether they engage shared or independent neurobiological pathways.

Pre-registration and transparent reporting. All future trials should be prospectively registered with clearly pre-specified primary endpoints and reported in accordance with CONSORT guidelines.

### 4.6. Strengths and Limitations of This Review

This systematic review has several strengths. It is, to our knowledge, the first to comprehensively evaluate the evidence for ambient or filtered green light interventions on pain and psychosocial outcomes using the RoB 2 and ROBINS-I tools with GRADE certainty assessment. The stratification by comparison type (green light vs. sham/no treatment and green light vs. other wavelengths), use of harvest plots, and analysis of delivery method and exposure parameters as potential effect modifiers provide a transparent and nuanced framework for interpreting the heterogeneous findings and guiding future trial design.

This review also has limitations. As OSF registration occurred after data extraction, this represents a deviation from best practice recommendations for prospective registration prior to study initiation. The search was limited to human studies published in English, which may have excluded relevant non-English literature. The heterogeneity of interventions, populations, and outcomes precluded meta-analysis, limiting the precision of effect estimates. Risk of bias assessments were conducted by a single reviewer and verified by a second, rather than being performed independently by two reviewers with subsequent reconciliation, which deviates from the Cochrane Handbook recommendation for independent dual assessment and may have introduced subjectivity into domain-level judgments, particularly for signaling questions requiring interpretation of study-specific methodological details [20]. The GRADE assessment was conducted narratively without pooled statistics, which introduces subjectivity into the rating of inconsistency and imprecision. Several included studies were not conventional parallel-group RCTs (e.g., within-subject repeated measures, contralateral-eye designs), requiring adaptation of the RoB 2 tool, which may not fully capture the unique biases of these designs. Finally, the small number of studies per outcome and comparison limited the ability to assess publication bias formally.

The heterogeneity of included studies should be viewed as both a limitation and a strength. As a limitation, it restricts the ability to draw population-specific conclusions, precludes meta-analytic pooling, and introduces uncertainty about whether observed effects are generalizable across clinical contexts. The diversity of outcome measures used across studies further limits direct comparability. As a strength, the inclusive approach provides a comprehensive evidence map that reveals the breadth of populations and outcomes for which green light therapy has been investigated, highlights consistent directional signals across diverse contexts, and identifies critical gaps to guide future research.

## 5. Conclusions

Across 13 studies encompassing approximately 668 participants, the majority of studies reported findings directionally favoring green light for pain and pain-related psychosocial outcomes, including the largest study with the lowest risk of bias. These consistent directional signals, combined with a plausible biological mechanism supported by preclinical evidence, suggest that green light therapy warrants continued investigation as a non-pharmaceutical intervention. However, the current evidence for green light therapy delivered as ambient or filtered exposure remains insufficient to support clinical recommendations for pain or psychosocial outcomes. The certainty of evidence remains very low across all outcomes, primarily due to the predominance of open-label designs that cannot distinguish specific from nonspecific effects. The current evidence is therefore best categorized as preliminary and hypothesis-generating rather than confirmatory. The field is well-positioned to move beyond pilot and feasibility studies toward adequately powered, pre-registered, randomized, sham-controlled trials with blinded outcome assessment, which will be essential to determine whether promising preliminary signals observed across diverse populations and clinical contexts reflect a true therapeutic effect of green light exposure.

## Figures and Tables

**Table 1:** Characteristics of randomized controlled trials of green light interventions.

References (Country, Year)	Condition Studied	No. Participants	Mean Years of Age ± SD (Range)	Intervention (Wavelength)	Comparator	Pain or Psychosocial Evaluation Tool
Takemura et al. [64] (Japan, 2025)	Dental Patients with Fear/Anxiety Related to IV Sedation	20	42.6 (36.2–48.2)	Green-colored goggles (530–547 nm); Worn for single session before and during PIC	Translucent, Blue (466–482 nm), Red (631–648 nm)	sAA activity *, HR, VAS Pain *, VAS treatment satisfaction
Kamata et al. [65] (Japan, 2025)	Migraine without aura	26	Migraine: 20.9 ± 0.7 Control: 21.4 ± 0.5	Green tinted lenses; Worn for 5 min during visual light stimulus	No lenses, Blue tinted lenses	Pupillometry (migraine *, control **), HRV, VAS for photophobia *
Mahmood et al. [66] (Pakistan, 2025)	Migraine	69	tDCS: 24.6 ± 6.3 sham tDCS: 23.8 ± 0.9 Green Light: 30.2 ± 11.8	Lamp emitting green light (525 ± 10 nm); Used for one hour per day for four weeks	Transcranial Direct Current Stimulation (tDCS), sham tDCS	Headache frequency *, NPS **, MPI **, Medication dependency *, Migraine-Specific Quality of Life **
Sawicki et al. [67] (United States, 2024)	Autism Spectrum Disorder	12	10.5 ± 2.2 (7–14)	Green light-exposed dental operatory exposure occurring during dental prophylaxis	White light-exposed dental operatory	HRV, Salivary cortisol, Salivary AMY1, FBRS, VBRS, VARS, FPS-R, r-FLACC
Gürses et al. [68] (Turkey, 2024)	Dental anxiety and intraoperative pain during third-molar extraction	128	24.25 ± 6.08 (18–40)	Green-colored glasses; Worn for single session before and during third-molar extraction	Clear Eyeglasses	STAI-S **, VAS for Dental Anxiety **, VAS for Pain *, Heart rate *, Blood pressure
Nelli et al. [69] (United States, 2023)	Fibromyalgia	34	57 ± 10	Green-light filtering eyeglasses worn at least 4 h per day for 2 weeks	Clear-light and Blue-light Filtering Eyeglasses	Opioid use, PROMIS-57, 11-Point NRS for Pain
Posternack et al. [70] (United States, 2023)	Episodic Migraine	78	46.2 (18–74)	Spectacles with filter that transmits green light wavelengths (500–570 nm); Worn as needed for 2–4 h post-migraine onset	Control Spectacles Blocked Less than 20% at 480 and 590 nm	11-Point NRS for Pain
Takemura et al. [71] (Japan, 2021)	Dental Patients with Fear/Anxiety Related to IV Sedation	24	40.9 ± 11.9	Green-colored glasses (532 nm); Worn for single session before and during PIC	Clear Eyeglasses	VAS for Anxiety, VAS for Pain *, sAA activity *, stress-related hemodynamic changes
Sharma et al. [72] (India, 2022)	Routine Fundus Evaluation for Myopia	100	25 ± 4.95 (18–40)	Green light filter used with binocular indirect ophthalmoscope during routine fundus evaluation	Yellow light filter	Level of comfort **, discomfort during exam **, and patient cooperation and duration of examination **

* Statistical significance was found for either the intervention or comparator with *p* < 0.05. ** Statistical significance was found for either the intervention or comparator with *p* < 0.001. Abbreviations: SD: Standard deviation; PIC: peripheral intravenous cannulation; sAA: Salivary alpha-amylase; HR: heart rate; VAS: Visual Analog Scale; HRV: heart rate variability; NPS: numeric pain scale; MPI: Multidimensional Pain Inventory; AMY1: amylase-1; FBRS: Frankl Behavior Rating Scale; VBRS: Venham Behavior Rating Scale; VARS: Venham Anxiety Rating Scale; FPS-R: Faces Pain Scale-Revised; r-FLACC: Revised-Face, Legs, Activity, Cry and Consolability Scale; STAI-S: State-Trait Anxiety Inventory–only state part; PROMIS: Patient-Reported Outcomes Measurement Information System; NRS: Numerical Rating Scale.

**Table 2:** Characteristics of non-randomized studies of green light interventions.

References (Country, Year)	Condition Studied	No. Participants	Mean Years of Age ± SD (Range)	Intervention (Wavelength)	Comparator	Pain or Psychosocial Evaluation Tool
Martin et al. [73] (United States, 2021)	Episodic migraine and chronic migraine	CM: 22 EM: 7	52.2 ± 3 (24–72)	2 h daily exposure to white LED for 10 weeks, 2-week washout, 1–2 h daily exposure to green LED (525 ± 10 nm) for 10 weeks	White LED	No. of headache days per month, NPS, EQ-5D-5L, HIT-6 for episodic migraine *, chronic migraine **, and all patients **
Martin et al. [74] (United States, 2021)	Fibromyalgia	21	53.25 ± 2.9 (26–75)	2 h daily exposure to white LED for 10 weeks, 2-week washout, 1–2 h daily exposure to green LED (525 ± 10 nm) for 10 weeks	White LED	NPS ***, SF MPQ *, FIQ **
Nir et al. [75] (United States, 2018)	Migraine Photophobia	Migraine: 69 † Healthy: 17	Migraine: 39 (29–49) Healthy: 44 (31–51)	Full-field ganzfeld ColorDome providing Green light exposure (530 ± 10 nm)	White, blue (447 ± 10), amber (590 ± 10), and red (627 ± 10) light exposure	Change in headache intensity during ictal phase, Onset of headache during interictal phase *
Noseda et al. [76] (United States, 2016)	Episodic Migraine Photophobia	41	40 ± 12 (15–85)	Full-field ganzfeld ColorDome providing Green light exposure (530 ± 10 nm)	White, blue (447 ± 10), amber (590 ± 10), and red (627 ± 10) light exposure	Verbal Analog Scale for Pain Intensity ***

* Statistical significance was found for either the intervention or comparator with *p* < 0.05. ** Statistical significance was found for either the intervention or comparator with *p* < 0.001. *** Statistical significance was found for either the intervention or comparator with *p* < 0.0001. Abbreviations: SD: Standard deviation; CM: chronic migraine; EM: episodic migraine; LED: light emitting diodes; NPS: numeric pain scale; EQ-5D-5L: EuroQol 5-Dimension 5-Level; HIT-6: Headache Impact Test-6; FIQ: Fibromyalgia Impact Questionnaire. † Noseda et al. (2016) and Nir et al. (2018) [75,76] originate from the same research group and share overlapping ictal migraine participant data. Ictal phase data originally collected for Noseda et al. [76] were incorporated into the Nir et al. [75] analysis. These studies are presented separately because they address distinct research questions (cone-driven photophobia pathways vs. color-selective photophobia across migraine phases), but the shared participants should be considered when interpreting the independence of findings.

**Table 3:** GRADE summary of findings: ambient/filtered green light interventions for pain and pain-related psychosocial outcomes in Comparison A: green light vs. sham/no treatment.

Outcome	No. of Studies (Participants)	Certainty	GRADE Informative Statement & Narrative Summary
Pain *	7 RCTs [64,66,67,68,69,70,71] + 4 NRSI [73,74,75,76] (~525 †)	⊕**◯◯◯** Very Low	The evidence is very uncertain about the effect of ambient/filtered green light on pain. Direction of Effect: ↔ Mixed.
Anxiety	3 RCTs [68,69,71] (186)	⊕**◯◯◯** Very Low	The evidence is very uncertain about the effect of ambient/filtered green light on anxiety. Direction of Effect: ↑ Favors green light.
Physiological Stress Markers	5 RCTs (210) [64,65,67,68,71]	⊕**◯◯◯** Very Low	The evidence is very uncertain about the effect of ambient/filtered green light on physiological stress markers. Direction of Effect: ↔ Mixed.
Photophobia/Light Sensitivity	2 RCT [65,70] + 2 NRSI [75,76] (~174 †)	⊕**◯◯◯** Very Low	The evidence is very uncertain about the effect of ambient/filtered green light on photophobia. Direction of Effect: ↔ Mixed.
Quality of Life	1 RCT [66] + 2 NRSIs [73,74] (119)	⊕**◯◯◯** Very Low	The evidence is very uncertain about the effect of ambient/filtered green light on quality of life. Direction of Effect: ↑ Favors green light.
Medication Use and Opioid Consumption	2 RCT [66,69] + 2 NRSI [73,74] (153)	⊕**◯◯◯** Very Low	The evidence is very uncertain about the effect of ambient/filtered green light on opioid consumption. Direction of Effect: ↔ Mixed.
Behavioral Cooperation	1 RCT [67] (12)	⊕**◯◯◯** Very Low	The evidence is very uncertain about the effect of ambient/filtered green light on behavioral cooperation compared. Direction of Effect: ↔ No significant difference.

Certainty ratings: ⊕**◯◯◯** = very low. Direction of effect symbols: ↑ = favors green light; ↔ = no significant difference or mixed results. † denotes potential participant overlap between studies. * Critical outcome. Studies with comparators spanning both sham/no treatment and other wavelengths [64,65,75,76]) contribute to both Comparison A and Comparison B based on the specific comparator arm evaluated.

**Table 4:** GRADE summary of findings: ambient/filtered green light interventions for pain and pain-related psychosocial outcomes in comparison B: green light vs. other wavelengths.

Outcome	No. of Studies (Participants)	Certainty	GRADE Informative Statement & Narrative Summary
Pain *	3 RCTs [64,69,72] + 2 NRSIs [75,76] (281 †)	⊕**◯◯◯** Very Low	The evidence is very uncertain about the effect of green light on pain compared to other wavelengths. Direction of Effect: ↔ Mixed.
Anxiety	1 RCT [69] (34)	⊕**◯◯◯** Very Low	The evidence is very uncertain about the effect on anxiety compared to other wavelengths. Direction of Effect: ↔ No significant difference.
Physiological Stress Markers	2 RCTs [64,65] (46)	⊕**◯◯◯** Very Low	The evidence is very uncertain about the effect on physiological stress markers compared to other wavelengths. Direction of Effect: ↔ Mixed.
Photophobia/Light Sensitivity	1 RCT [65] + 2 NRSIs [75,76] (120 †)	⊕**◯◯◯** Very Low	The evidence is very uncertain about the effect on photophobia compared to other wavelengths. Direction of Effect: ↑ Favors green light.
Opioid Consumption	1 RCT [69] (34)	⊕**◯◯◯** Very Low	The evidence is very uncertain about the effect on opioid consumption compared to other wavelengths. Direction of Effect: ↔ No significant difference.
Patient Comfort/Cooperation	1 RCT [72] (100)	⊕**◯◯◯** Very Low	The evidence is very uncertain about the effect on patient comfort or cooperation compared to yellow light. Direction of Effect: ↑ Favors green light.

Certainty ratings: ⊕**◯◯◯** = very low. Direction of effect symbols: ↑ = favors green light; ↔ = no significant difference or mixed results. † denotes potential participant overlap between studies. * Critical outcome. Studies with comparators spanning both sham/no treatment and other wavelengths [64,65,75,76]) contribute to both Comparison A and Comparison B based on the specific comparator arm evaluated.

## Data Availability

This systematic review analyzed data from previously published studies; no new primary data were generated. All data supporting the findings of this review are available within the article and its Supplementary Materials. The full search strategies are provided in Appendix A. The study selection records, extracted data, and risk of bias assessments are available from the corresponding author upon reasonable request. The review protocol is registered with the Open Science Framework (OSF registration DOI/URL).

## Supplementary Materials

The following supporting information can be downloaded at: https://www.mdpi.com/article/10.3390/jcm15145710/s1.

## Abbreviations

The following abbreviations are used in this manuscript:

AMY1	Amylase-1
ASD	Autism Spectrum Disorder
CI	Confidence Interval
CINAHL	Cumulative Index of Nursing and Allied Health Literature
CM	Chronic Migraine
CONSORT	Consolidated Standards of Reporting Trials
DAS	Dental Anxiety Scale
DBP	Diastolic Blood Pressure
EM	Episodic Migraine
EQ-5D-5L	EuroQol 5-Dimension 5-Level
FBRS	Frankl Behavior Rating Scale
FIQ	Fibromyalgia Impact Questionnaire
FPS-R	Faces Pain Scale-Revised
GLED	Green Light-Emitting Diode
GRADE	Grading of Recommendations Assessment, Development and Evaluation
HFnu	Normalized High-Frequency Power
HIT-6	Headache Impact Test-6
HR	Heart Rate
HRV	Heart Rate Variability
IMMPACT	Initiative on Methods, Measurement, and Pain Assessment in Clinical Trials
ipRGC	Intrinsically Photosensitive Retinal Ganglion Cell
ITT	Intention-to-Treat
LED	Light-Emitting Diode
LFnu	Normalized Low-Frequency Power
MBP	Mean Blood Pressure
MPI	Multidimensional Pain Inventory
NPS	Numeric Pain Scale
NRS	Numerical Rating Scale
NRSI	Non-Randomized Study of Interventions
NS	Not significant
OME	Oral Morphine Equivalents
OSF	Open Science Framework
PICOS	Population, Intervention, Comparator, Outcomes, Study Design
PRISMA	Preferred Reporting Items for Systematic Reviews and Meta-Analyses
PROMIS	Patient-Reported Outcomes Measurement Information System
RCT	Randomized Controlled Trial
r-FLACC	Revised-Face, Legs, Activity, Cry and Consolability Scale
RoB 2	Cochrane Risk of Bias 2
ROBINS-I	Risk of Bias in Non-Randomized Studies of Interventions
sAA	Salivary Alpha-Amylase
SBP	Systolic Blood Pressure
SD	Standard Deviation
SF MPQ	Short-Form McGill Pain Questionnaire
STAI-S	State-Trait Anxiety Inventory–State Subscale
SWiM	Synthesis Without Meta-Analysis
tDCS	Transcranial Direct Current Stimulation
VARS	Venham Anxiety Rating Scale
VAS	Visual Analog Scale
VBRS	Venham Behavior Rating Scale
vLGN	ventrolateral geniculate nucleus
WLED	White Light-Emitting Diode

## Appendix A

### Database Search Strategies

**Table A1:** PubMed (NIH/NLM): 31 October 2025.

Search	Query	Number of Results
1	(“green light”[TIAB] OR green fluorescence[TIAB] OR green-light[TIAB] OR green phototherapy[TIAB] OR green fluorescent[TIAB] OR “550 nm”[TIAB] OR “green laser”[TIAB] OR “green lasers”[TIAB] OR green lights[TIAB] OR green lighted[TIAB] OR green lighting[TIAB] OR GLED[TIAB] OR green photobiomodulation[TIAB] OR green LED[TIAB] OR “520 nm”[TIAB] OR “550 nanometers”[TIAB] OR “520 nanometers”[TIAB] OR green emitting[TIAB] OR green-emitting[TIAB] OR “500 nm”[TIAB] OR “505 nm”[TIAB] OR “532 nm”[TIAB] OR “540 nm”[TIAB] OR “560 nm”[TIAB] OR “500 nanometers”[TIAB] OR “505 nanometers”[TIAB] OR “532 nanometers”[TIAB] OR “540 nanometers”[TIAB] OR “560 nanometers”[TIAB] OR “Green Light”[MeSH])	77,561
2	(pain[TIAB] OR pains[TIAB] OR painful[TIAB] OR migraine[TIAB] OR migraines[TIAB] OR headache[TIAB] OR headaches[TIAB] OR fibromyalgia[TIAB] OR analgesia[TIAB] OR osteoarthritis[TIAB] OR arthritis[TIAB] OR arthralgia[TIAB] OR myalgia[TIAB] OR antinociception [TIAB] OR antinociceptive[TIAB] OR nociceptive[TIAB] OR nociception [TIAB] OR “Pain”[MeSH] OR “Analgesia”[MeSH] OR “Pain Management”[MeSH] OR “Fibromyalgia”[MeSH] OR “Headache Disorders”[MeSH] OR “Arthritis”[MeSH])	1,627,175
3	#1 AND #2	804
4	(“Animals”[Mesh] NOT “Humans”[Mesh])	5,389,402
5	#3 NOT #4	491
6	(mice[Title] OR mouse[Title] OR rat[Title] OR rats[Title] OR review[Title] OR reviews[Title])	2,415,435
7	#5 NOT #6	436
8	Comment [Publication Type] OR Editorial [Publication Type] OR Review [Publication Type]	5,300,993
9	#7 NOT #8	414
10	#9 AND English[Filter]	402
Total		402

**Table A2:** Scopus (Elsevier): 31 October 2025.

Search	Query	Number of Results
1	(TITLE-ABS(“green light”) OR TITLE-ABS(green fluorescence) OR TITLE-ABS(green-light) OR TITLE-ABS(green phototherapy) OR TITLE-ABS(green fluorescent) OR TITLE-ABS(“550 nm”) OR TITLE-ABS(“green laser”) OR TITLE-ABS(“green lasers”) OR TITLE-ABS(green lights) OR TITLE-ABS(green lighted) OR TITLE-ABS(green lighting) OR TITLE-ABS(GLED) OR TITLE-ABS(green photobiomodulation) OR TITLE-ABS(green LED) OR TITLE-ABS(“520 nm”) OR TITLE-ABS(“550 nanometers”) OR TITLE-ABS(“520 nanometers”) OR TITLE-ABS(green emitting) OR TITLE-ABS(green-emitting) OR TITLE-ABS(“500 nm”) OR TITLE-ABS(“505 nm”) OR TITLE-ABS(“532 nm”) OR TITLE-ABS(“540 nm”) OR TITLE-ABS(“560 nm”) OR TITLE-ABS(“500 nanometers”) OR TITLE-ABS(“505 nanometers”) OR TITLE-ABS(“532 nanometers”) OR TITLE-ABS(“540 nanometers”) OR TITLE-ABS(“560 nanometers”))	291,798
2	(TITLE-ABS(pain) OR TITLE-ABS(pains) OR TITLE-ABS(painful) OR TITLE-ABS(migraine) OR TITLE-ABS(migraines) OR TITLE-ABS(headache) OR TITLE-ABS(headaches) OR TITLE-ABS(fibromyalgia) OR TITLE-ABS(analgesia) OR TITLE-ABS(osteoarthritis) OR TITLE-ABS(arthritis) OR TITLE-ABS(arthralgia) OR TITLE-ABS(myalgia) OR TITLE-ABS(antinociception) OR TITLE-ABS(antinociceptive) OR TITLE-ABS(nociceptive) OR TITLE-ABS(nociception))	1,685,712
3	#1 AND #2	1309
4	#3 AND (EXCLUDE (DOCTYPE,”ed”) OR EXCLUDE (DOCTYPE,”no”) OR EXCLUDE (DOCTYPE,”cp”) OR EXCLUDE (DOCTYPE,”re”))	1208
5	TITLE (mice OR mouse OR rat OR rats OR review OR reviews)	3,273,114
6	#5 AND NOT #6	977
7	#6 AND (LIMIT-TO (LANGUAGE,”English”))	914
Total		914

**Table A3:** Embase (Elsevier): 31 October 2025.

Search	Query	Number of Results
1	(‘green light’:ti,ab,kw OR ‘green fluorescence’:ti,ab,kw OR ‘green-light’:ti,ab,kw OR ‘green phototherapy’:ti,ab,kw OR ‘green fluorescent’:ti,ab,kw OR ‘550 nm’:ti,ab,kw OR ‘green laser’:ti,ab,kw OR ‘green lasers’:ti,ab,kw OR ‘green lights’:ti,ab,kw OR ‘green lighted’:ti,ab,kw OR ‘green lighting’:ti,ab,kw OR ‘gled’:ti,ab,kw OR ‘green photobiomodulation’:ti,ab,kw OR ‘green led’:ti,ab,kw OR ‘520 nm’:ti,ab,kw OR ‘550 nanometers’:ti,ab,kw OR ‘520 nanometers’:ti,ab,kw OR ‘green emitting’:ti,ab,kw OR ‘green-emitting’:ti,ab,kw OR ‘500 nm’:ti,ab,kw OR ‘505 nm’:ti,ab,kw OR ‘532 nm’:ti,ab,kw OR ‘540 nm’:ti,ab,kw OR ‘560 nm’:ti,ab,kw OR ‘500 nanometers’:ti,ab,kw OR ‘505 nanometers’:ti,ab,kw OR ‘532 nanometers’:ti,ab,kw OR ‘540 nanometers’:ti,ab,kw OR ‘560 nanometers’:ti,ab,kw OR ‘green light’/exp)	87,317
2	‘pain’:ti,ab,kw OR ‘pains’:ti,ab,kw OR ‘painful’:ti,ab,kw OR ‘migraine’:ti,ab,kw OR ‘migraines’:ti,ab,kw OR ‘headache’:ti,ab,kw OR ‘headaches’:ti,ab,kw OR ‘fibromyalgia’:ti,ab,kw OR ‘analgesia’:ti,ab,kw OR ‘osteoarthritis’:ti,ab,kw OR ‘arthritis’:ti,ab,kw OR ‘arthralgia’:ti,ab,kw OR ‘myalgia’:ti,ab,kw OR ‘antinociception’:ti,ab,kw OR ‘antinociceptive’:ti,ab,kw OR ‘nociceptive’:ti,ab,kw OR ‘nociception’:ti,ab,kw OR ‘pain’/exp OR ‘analgesia’/exp OR ‘fibromyalgia’/exp OR ‘headache and facial pain’/exp OR ‘arthritis’/exp	3,128,772
3	#1 AND #2	1532
4	#3 NOT (‘conference abstract’/it OR ‘conference paper’/it OR ‘conference review’/it OR ‘editorial’/it OR ‘letter’/it OR ‘note’/it OR ‘review’/it)	1018
5	#4 NOT ([animals]/lim NOT [humans]/lim)	624
6	#5 AND [english]/lim	605
7	#5 NOT (rats:ti OR rat:ti OR mouse:ti OR mice:ti OR review:ti OR reviews:ti)	579
Total		579

**Table A4:** CINAHL (EBSCOhost): 31 October 2025.

Search	Query	Number of Results
1	(XB “green light” OR XB green fluorescence OR XB green-light OR XB green phototherapy OR XB green fluorescent OR XB “550 nm” OR XB “green laser” OR XB “green lasers” OR XB green lights OR XB green lighted OR XB green lighting OR XB GLED OR XB green photobiomodulation OR XB green LED OR XB “520 nm” OR XB “550 nanometers” OR XB “520 nanometers” OR XB green emitting OR XB green-emitting OR XB “500 nm” OR XB “505 nm” OR XB “532 nm” OR XB “540 nm” OR XB “560 nm” OR XB “500 nanometers” OR XB “505 nanometers” OR XB “532 nanometers” OR XB “540 nanometers” OR XB “560 nanometers” OR DE “Green Light”)	2666
2	(XB pain OR XB pains OR XB painful OR XB migraine OR XB migraines OR XB headache OR XB headaches OR XB fibromyalgia OR XB analgesia OR XB osteoarthritis OR XB arthritis OR XB arthralgia OR XB myalgia OR XB antinociception OR XB antinociceptive OR XB nociceptive OR XB nociception OR MH “Pain+” OR MH “Analgesia+” OR MH “Pain Management+” OR DE “Fibromyalgia” OR DE “Migraine” OR MH “Arthritis+”)	548,286
3	#1 AND #2	133
4	#3 NOT TI (rat OR rats OR mouse OR mice OR review OR reviews)	100
5	#4 AND Filters: English Language and Academic Journals	93
Total		93

**Table A5:** PsycINFO (EBSCOhost): 31 October 2025.

Search	Query	Number of Results
1	((TI “green light” OR AB “green light”) OR (TI green fluorescence OR AB green fluorescence) OR (TI green-light OR AB green-light) OR (TI green phototherapy OR AB green phototherapy) OR (TI green fluorescent OR AB green fluorescent) OR (TI “550 nm” OR AB “550 nm”) OR (TI “green laser” OR AB “green laser”) OR (TI “green lasers” OR AB “green lasers”) OR (TI green lights OR AB green lights) OR (TI green lighted OR AB green lighted) OR (TI green lighting OR AB green lighting) OR (TI GLED OR AB GLED) OR (TI green photobiomodulation OR AB green photobiomodulation) OR (TI green LED OR AB green LED) OR (TI “520 nm” OR AB “520 nm”) OR (TI “550 nanometers” OR AB “550 nanometers”) OR (TI “520 nanometers” OR AB “520 nanometers”) OR (TI green emitting OR AB green emitting) OR (TI green-emitting OR AB green-emitting) OR (TI “500 nm” OR AB “500 nm”) OR (TI “505 nm” OR AB “505 nm”) OR (TI “532 nm” OR AB “532 nm”) OR (TI “540 nm” OR AB “540 nm”) OR (TI “560 nm” OR AB “560 nm”) OR (TI “500 nanometers” OR AB “500 nanometers”) OR (TI “505 nanometers” OR AB “505 nanometers”) OR (TI “532 nanometers” OR AB “532 nanometers”) OR (TI “540 nanometers” OR AB “540 nanometers”) OR (TI “560 nanometers” OR AB “560 nanometers”))	2851
2	((TI pain OR AB pain) OR (TI pains OR AB pains) OR (TI painful OR AB painful) OR (TI migraine OR AB migraine) OR (TI migraines OR AB migraines) OR (TI headache OR AB headache) OR (TI headaches OR AB headaches) OR (TI fibromyalgia OR AB fibromyalgia) OR (TI analgesia OR AB analgesia) OR (TI osteoarthritis OR AB osteoarthritis) OR (TI arthritis OR AB arthritis) OR (TI arthralgia OR AB arthralgia) OR (TI myalgia OR AB myalgia) OR (TI antinociception OR AB antinociception) OR (TI antinociceptive OR AB antinociceptive) OR (TI nociceptive OR AB nociceptive) OR (TI nociception OR AB nociception) OR DE “Pain” OR DE “Analgesia” OR DE “Pain Management” OR DE “Fibromyalgia” OR DE “Migraine Headache” OR DE “Arthritis”)	158,930
3	#1 AND #2	94
4	#3 NOT TI (rat OR rats OR mouse OR mice OR review OR reviews)	56
5	#4 AND Filters: English Language and Academic Journals	52
Total		52
