# Supplementary material for: Green Light Therapy for Pain and Pain-Related Psychosocial Outcomes: A Systematic Review of Human Studies
Source: J Clin Med. 2026 Jul 21;15(14):5710. doi: 10.3390/jcm15145710 (PMC13412275; doi:10.3390/jcm15145710)
Supplement: Supplementary file 1 [file jcm-15-05710-s001.zip › jcm-4414080-supplementary.pdf]

Supplementary Materials

Table S1. PRISMA 2020 Checklist

Section and Topic	Item #	Checklist item	Location where item is reported
**TITLE**			
Title	1	Identify the report as a systematic review.	Title, page 1
**ABSTRACT**			
Abstract	2	See the PRISMA 2020 for Abstracts checklist.	Abstract, page 1
**INTRODUCTION**			
Rationale	3	Describe the rationale for the review in the context of existing knowledge.	Section 1, page 1-2
Objectives	4	Provide an explicit statement of the objective(s) or question(s) the review addresses.	Section 1, page 1-2
**METHODS**			
Eligibility criteria	5	Specify the inclusion and exclusion criteria for the review and how studies were grouped for the syntheses.	Section 2.2, page 3-4; Section 2.7, page 6
Information sources	6	Specify all databases, registers, websites, organisations, reference lists and other sources searched or consulted to identify studies. Specify the date when each source was last searched or consulted.	Section 2.1, page 3
Search strategy	7	Present the full search strategies for all databases, registers and websites, including any filters and limits used.	Appendix A, Tables A1-A5, pages 30-34
Selection process	8	Specify the methods used to decide whether a study met the inclusion criteria of the review, including how many reviewers screened each record and each report retrieved, whether they worked independently, and if applicable, details of automation tools used in the process.	Section 2.3, page 4
Data collection process	9	Specify the methods used to collect data from reports, including how many reviewers collected data from each report, whether they worked independently, any processes for obtaining or confirming data from study investigators, and if applicable, details of automation tools used in the process.	Section 2.4, page 4-5
Data items	10a	List and define all outcomes for which data were sought. Specify whether all results that were compatible with each outcome domain in each study were sought (e.g. for all measures, time points, analyses), and if not, the methods used to decide which results to collect.	Section 2.2.4, page 3; Section 2.4, page 4-5
	10b	List and define all other variables for which data were sought (e.g. participant and intervention characteristics, funding sources). Describe any assumptions made about any missing or unclear information.	Section 2.4, page 4-5
Study risk of bias assessment	11	Specify the methods used to assess risk of bias in the included studies, including details of the tool(s) used, how many reviewers assessed each study and whether they worked independently, and if applicable, details of automation tools used in the process.	Section 2.5, page 5
Effect measures	12	Specify for each outcome the effect measure(s) (e.g. risk ratio, mean difference) used in the synthesis or presentation of results.	Section 2.7, page 6; Section 2.6, page 5-6; Section 3.4, page 12
Synthesis methods	13a	Describe the processes used to decide which studies were eligible for each synthesis (e.g. tabulating the study intervention characteristics and comparing against the planned groups for each synthesis (item #5)).	Section 2.6, page 5-6; Section 2.7, page 6
	13b	Describe any methods required to prepare the data for presentation or synthesis, such as handling of missing summary statistics, or data conversions.	Section 2.4, page 4-5

	13c	Describe any methods used to tabulate or visually display results of individual studies and syntheses.	Section 2.7, page 6
	13d	Describe any methods used to synthesize results and provide a rationale for the choice(s). If meta-analysis was performed, describe the model(s), method(s) to identify the presence and extent of statistical heterogeneity, and software package(s) used.	Section 2.7, page 6
	13e	Describe any methods used to explore possible causes of heterogeneity among study results (e.g. subgroup analysis, meta-regression).	Section 2.6, page - 6;<br>Section 2.7, page 6;<br>Section 4.2, page 24-25
	13f	Describe any sensitivity analyses conducted to assess robustness of the synthesized results.	Not applicable
Reporting bias assessment	14	Describe any methods used to assess risk of bias due to missing results in a synthesis (arising from reporting biases).	Section 2.5, page 5;<br>Section 3.6, page 19 -20
Certainty assessment	15	Describe any methods used to assess certainty (or confidence) in the body of evidence for an outcome.	Section 2.6, page 5-6
**RESULTS**			
Study selection	16a	Describe the results of the search and selection process, from the number of records identified in the search to the number of studies included in the review, ideally using a flow diagram.	Section 3.1, page 6;<br>Figure 1, page 7
	16b	Cite studies that might appear to meet the inclusion criteria, but which were excluded, and explain why they were excluded. I included how many were excluded but did not cite them.	Section 3.1, page 6;<br>Figure 1, page 7;<br>Section 3.2.4, page 11
Study characteristics	17	Cite each included study and present its characteristics.	Section 3.2, page 7-11; Tables 1 and 2, page 7-9
Risk of bias in studies	18	Present assessments of risk of bias for each included study.	Section 3.3, page 11-12; Figure S1; Figure S2
Results of individual studies	19	For all outcomes, present, for each study: (a) summary statistics for each group (where appropriate) and (b) an effect estimate and its precision (e.g. confidence/credible interval), ideally using structured tables or plots.	Section 3.4, page 12-17
Results of syntheses	20a	For each synthesis, briefly summarise the characteristics and risk of bias among contributing studies.	Section 3.8, page 19-22; Figure 2, page 20-21
	20b	Present results of all statistical syntheses conducted. If meta-analysis was done, present for each the summary estimate and its precision (e.g. confidence/credible interval) and measures of statistical heterogeneity. If comparing groups, describe the direction of the effect.	Section 3.8, page 19-22; Figure 2, page 20-21
	20c	Present results of all investigations of possible causes of heterogeneity among study results.	Section 3.8.1-3.8.3, page 21-22; Section 4.2, page 24-25
	20d	Present results of all sensitivity analyses conducted to assess the robustness of the synthesized results.	Not applicable
Reporting biases	21	Present assessments of risk of bias due to missing results (arising from reporting biases) for each synthesis assessed.	Section 3.7, page 17-19; Tables S4 and S5
Certainty of evidence	22	Present assessments of certainty (or confidence) in the body of evidence for each outcome assessed.	Section 3.7, page 17-19; Tables 3 and 4, page 17-19; Tables S4 and S5

**DISCUSSION**			
Discussion	23a	Provide a general interpretation of the results in the context of other evidence.	Section 4.1, page 23; Section 4.4, page 27
	23b	Discuss any limitations of the evidence included in the review.	Section 4.3, page 26
	23c	Discuss any limitations of the review processes used.	Section 4.6, page 27-28
	23d	Discuss implications of the results for practice, policy, and future research.	Section 4.5, page 27
**OTHER INFORMATION**			
Registration and protocol	24a	Provide registration information for the review, including register name and registration number, or state that the review was not registered.	Section 2.1, page 3
	24b	Indicate where the review protocol can be accessed, or state that a protocol was not prepared.	Section 2.1, page 3
	24c	Describe and explain any amendments to information provided at registration or in the protocol.	Section 2.1, page 3
Support	25	Describe sources of financial or non-financial support for the review, and the role of the funders or sponsors in the review.	Page 29
Competing interests	26	Declare any competing interests of review authors.	Page 29
Availability of data, code and other materials	27	Report which of the following are publicly available and where they can be found: template data collection forms; data extracted from included studies; data used for all analyses; analytic code; any other materials used in the review.	Page 29

Table S2. Characteristics of excluded studies

Study	Reason for exclusion
Abdallah et al., 2023 [27]	Wrong intervention: fluorescence assay of human plasma; not a green light therapy intervention
Adam et al., 2018 [28]	Wrong intervention: laser photocoagulation targeting retinal tissue, not green light therapy via visual pathways
Áfra et al., 2000 [29]	Wrong population: exclusively healthy volunteers
Amato et al., 2024 [30]	Wrong study design: narrative article
Bargiela-Pérez et al., 2018 [31]	Wrong intervention: surgical laser used for resection of oral lesions, not green light therapy via visual pathways
Baron et al., 2024 [32]	Wrong intervention: blue light was the primary intervention; green light served as sham
Chen et al., 2025 [33]	Wrong population: animal study
Cheng et al., 2022 [34]	Wrong study design: case report
Colloca et al., 2009 [35]	Wrong population: exclusively healthy volunteers
Eroğlu et al., 2024 [36]	Wrong intervention: photodynamic therapy with photosensitizer applied to tissue; not within 495–570 nm green light range
Flor et al., 1999 [37]	Wrong population: exclusively healthy volunteers
Fornaini et al., 2012 [38]	Wrong study design: no control intervention
Fritsch et al., 1997 [39]	Wrong intervention: photodynamic therapy applied to skin tissue, not green light therapy via visual pathways
González-Saldivar et al., 2017 [40]	Wrong intervention: laser photocoagulation targeting retinal tissue, not green light therapy via visual pathways
Harle et al., 2006 [41]	Wrong intervention: did not isolate green light as a distinct intervention
Hou et al., 2024 [42]	Wrong study design: review article
Hu et al., 2021 [43]	Wrong intervention: low-level laser therapy applied topically to tissue, not green light therapy via visual pathways
Ismail et al., 2025 [18]	Wrong population: animal study
Jamieson, 2016 [44]	Wrong study design: Commentary
Karran et al., 2018 [45]	Wrong outcome: did not assess pain intensity or pain-related psychosocial outcomes
Landgrebe et al., 2008 [46]	Wrong population: exclusively healthy volunteers
Li et al., 2020 [47]	Wrong study design: no control intervention
Lipton et al., 2023 [48]	Wrong study design: uncontrolled open-label study
Lira et al., 2010 [49]	Wrong intervention: laser photocoagulation targeting retinal tissue, not green light therapy via visual pathways
Mahroo, 2017 [50]	Wrong study design: Peer-reviewed commentary
Martin et al., 2021 [51]	Wrong population: animal study
Martin et al., 2023 [15]	Wrong population: animal study

Study	Reason for exclusion
Morton et al., 2000 [52]	Wrong outcome: insufficient extractable pain data for quantitative analysis
Nagpal et al., 2010 [53]	Wrong intervention: laser photocoagulation targeting retinal tissue, not green light therapy via visual pathways
Oren et al., 1991 [54]	Wrong outcome: no pain or pain-related psychosocial outcome reported
Osiecka et al., 2017 [55]	Wrong study design: no control intervention
Osiecka et al., 2018 [56]	Wrong intervention: photodynamic therapy applied to skin tissue, not green light therapy via visual pathways
Serrage et al., 2019 [57]	Wrong study design: review article
Seymenoğlu et al., 2013 [58]	Not available in English
Topics in Pain Management, 2020 [59]	Wrong study design: Commentary
Ventura et al., 2024 [60]	Wrong population: animal study
Wiercioch-Kuzianik et al., 2019 [61]	Healthy subjects<br>Wrong patient population
Wilkins et al., 2021 [62]	Wrong study design: case report
Wu et al., 2023 [63]	Wrong population: animal study

Table S3. Registered clinical trials awaiting classification (study not completed)

ClinicalTrials.gov Registration	Status
NCT00155584	Recruiting (registered 2005)
NCT01044875	Recruiting (registered 2009)
NCT03674697	Active, not recruiting
NCT03677206	Active, not recruiting
NCT03857074	Unknown; publication not found
NCT04256915	Not yet started
NCT05295225	Active, not recruiting
NCT05398666	Active, not recruiting
NCT05561270	Recruiting
NCT05569486	Not yet started
NCT05763381	Completed; publication not found
NCT05853731	Withdrawn
NCT05956067	Paused
NCT06161129	Not yet started

ClinicalTrials.gov Registration	Status
NCT06395584	Ongoing
NCT06772519	Completed; publication not found
NCT06943625	Recruiting
NCT07033949	Not yet started

Outcome	Study	D1	D2	D3	D4	D5	DS	Overall
Pain	Mahmood et al. 2025	–	✖	✖	✖	–	NA	✖
	Sawicki et al. 2024	–	–	–	–	+	–	–
	Takemura et al. 2025*	+	–	+	–	–	+	–
	Gürses et al. 2024	✖	–	+	–	+	NA	–
	Nelli et al. 2023	+	✖	✖	–	+	NA	✖
	Posternack et al. 2023	+	–	–	+	+	NA	–
	Posternack et al. 2023 (post-hoc)	–	–	–	+	✖	NA	✖
	Takemura et al. 2021	–	–	+	✖	–	–	✖
	Sharma et al. 2022	✖	–	+	✖	–	NA	✖
Anxiety	Sawicki et al. 2024	–	–	–	–	+	–	–
	Gürses et al. 2024	✖	–	+	–	+	NA	–
	Nelli et al. 2023	+	✖	✖	–	–	NA	✖
	Takemura et al. 2021	–	–	–	✖	–	–	✖
Physiological Stress Markers	Sawicki et al. 2024	–	–	–	+	+	–	–
	Kamata et al. 2025	–	–	+	+	–	–	–
	Takemura et al. 2025*	+	–	+	+	–	+	–
	Gürses et al. 2024	✖	–	+	–	+	NA	–
	Takemura et al. 2021	–	–	+	+	–	–	–
Photophobia / Light Sensitivity	Kamata et al. 2025	–	–	+	–	–	–	–
	Posternack et al. 2023	+	–	–	–	+	NA	–
	Posternack et al. 2023 (post-hoc)	–	–	–	–	✖	NA	–
Quality of Life	Mahmood et al. 2025	–	✖	✖	✖	–	NA	✖
Medication Use and Opioid Consumption	Mahmood et al. 2025	–	✖	✖	–	–	NA	✖
	Nelli et al. 2023	+	–	✖	+	+	NA	✖
Behavioral Cooperation	Sawicki et al. 2024	–	–	–	–	+	–	–
Patient Comfort/Cooperation	Sharma et al. 2022	✖	–	+	✖	–	NA	✖

+ Low risk
– Some concerns
✖ High risk

Ambient green light exposure
Green-tinted / wave-length filtering lenses
Red-free (green) examination light

* Takemura et al. 2025 self-identifies as a "prospective observational study" with a within-subject repeated measures design, not a randomized controlled trial. The study does incorporate three-arm intervention and randomization (Latin square) of the order of conditions, therefore the study was reclassified as a randomized crossover trial for the purposes of this review.

Figure S1. Risk of bias assessment using the Cochrane Risk of Bias 2 (RoB 2) tool.
Domain-level and overall risk of bias judgments for outcomes in all nine included randomized studies [27-35] categorized by intervention delivery method. Domains assessed: D1 = bias arising from the randomization process; D2 = bias due to deviations from intended interventions; D3 = bias due to missing outcome data; D4 = bias in measurement of the outcome; D5 = bias in selection of the re-ported result, DS = bias arising from period and carryover effects. No study received an overall low risk of bias judgment for any outcome. Five of nine ambient/filtered green light studies were judged as overall high risk of bias for the primary outcome of pain.

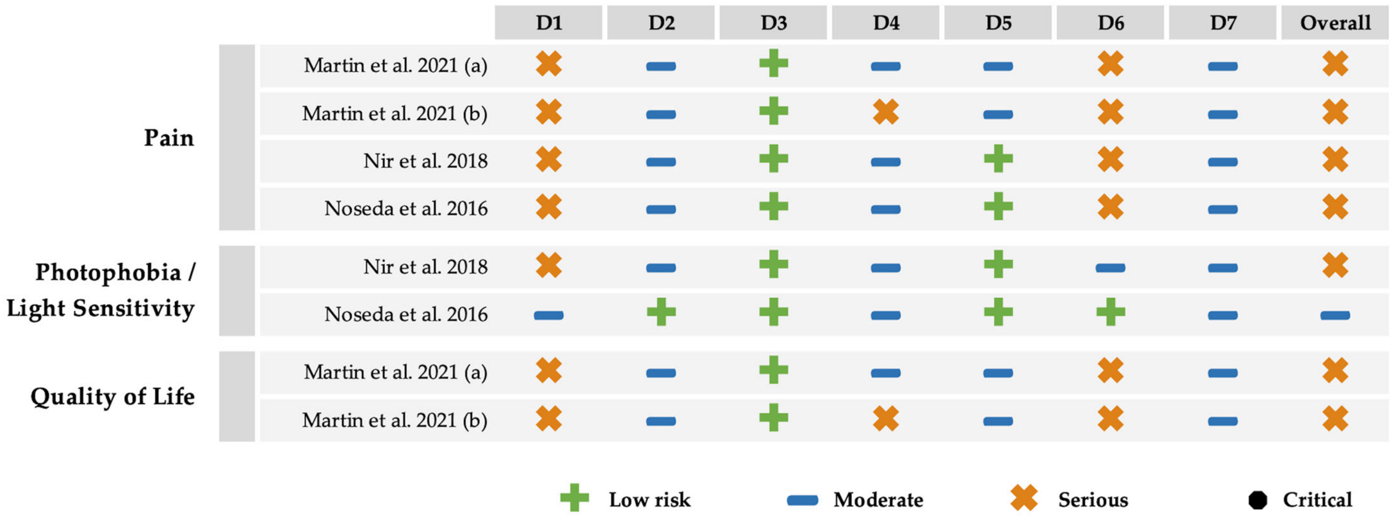

Figure S2. Risk of bias assessment using the Risk of Bias in Non-Randomized Studies of Interventions (ROBINS-I) tool. Domain-level and overall risk of bias judgments for all four included non-randomized studies [36-39] across three outcomes. Domains assessed: D1 = bias due to confounding; D2 = bias in selection of participants into the study; D3 = bias in classification of interventions; D4 = bias due to deviations from intended interventions; D5 = bias due to missing data; D6 = bias in measurement of outcomes; D7 = bias in selection of reported result. No study received an overall low risk of bias judgment. All outcomes assessed in the four ambient/filtered green light studies were judged as overall serious risk of bias, with only one study being judged as moderate for photophobia/light sensitivity. The most pervasive source of bias was Domain 1.

Table S4. GRADE Evidence Profile: Ambient/Filtered Green Light vs. Sham/No Treatment (Comparison A)

Outcome	No. of Studies (Participants)	Risk of Bias	Inconsistency	Indirectness	Imprecision	Publication Bias	Certainty
Pain	7 RCTs [27,29-34] + 4 NRSI [36-39] (~525†)	Very serious (−2): universal; see text	Serious (−1): only double-blind trial failed pre-specified endpoints; positive findings limited to open-label designs and post-hoc analyses	Serious (−1): heterogeneous populations, delivery methods, and exposure durations	Serious (−1): median n = 34; most studies lacked a priori power calculations for pain; wide CIs where reported	Serious (−1): With 7 RCTs, formal funnel plot analysis falls below the 10-study threshold and cannot be performed. Qualitative indicators strongly suggest publication bias (small sample size, overlapping authorship, lack of pre-registration in several studies)	⨁◯◯◯ Very Low
Anxiety	3 RCTs [31,32,34] (186)	Very serious (−2): universal; see text	Not serious: all three studies favored green light, though magnitude and significance varied	Serious (−1): conditions investigated represent different constructs with different instruments	Serious (−1): only one study adequately sized; PROMIS finding emerged from one of seven domains without multiplicity correction	Not downgraded: Formal assessment not possible. Qualitative concerns present but insufficient to formally downgrade.	⨁◯◯◯ Very Low
Physiological Stress Markers	5 RCTs (210) [27,28,30,31,34]	Serious (−1): universal; see text	Serious (−1): significant sAA in 2 RCTs but hemo-	Serious (−1): different stress markers (sAA, HR,	Serious (−1): samples 12–128; most not pow-	Not downgraded: Formal assessment not possi-	⨁◯◯◯ Very Low

Outcome	No. of Studies (Participants)	Risk of Bias	Inconsistency	Indirectness	Imprecision	Publication Bias	Certainty
			dynamic measures predominantly null; null HRV/cortisol; HRV lacked formal interaction test	HRV, cortisol, BP) across different clinical contexts	ered for physiological outcomes	ble. Qualitative concerns present but insufficient to formally downgrade.	
Photophobia /Light Sensitivity	2 RCT [28,33] + 2 NRSI [38,39] (~174†)	Very serious (−2): universal; see text	Serious (−1): one RCT post-hoc positive only; one showed no normalization of VAS photophobia; NRSIs consistent but non-independent	Serious (−1): episodic migraine subgroup analysis; laboratory setting; perceptual paradigm	Not serious: larger combined sample, though non-independence limits effective n	Not downgraded: Formal assessment not possible. Qualitative concerns present but insufficient to formally downgrade.	⨁◯◯◯ Very Low
Quality of Life	1 RCT [29] + 2 NRSIs [36,37] (119)	Very serious (−2): universal; see text	Not serious: all three studies favored green light	Serious (−1): different QoL instruments across different populations and comparators	Serious (−1): small samples; NRSIs lacked a priori power calculations for QoL	Not downgraded: Formal assessment not possible. Qualitative concerns present but insufficient to formally downgrade.	⨁◯◯◯ Very Low
Medication Use and Opioid Consumption	2 RCT [29,32] + 2 NRSI [36,37] (153)	Very serious (−2): Self-reported medication use by unblinded participants	Serious (−1): no study demonstrated significant effect on a pre-specified medication endpoint	Serious (−1): fundamentally different medication outcomes across different populations	Serious (−1): small samples; no study powered for medication use; feasibility pilot	Not downgraded: Formal assessment not possible. Qualitative concerns present but insufficient to formally	⨁◯◯◯ Very Low

Outcome	No. of Studies (Participants)	Risk of Bias	Inconsistency	Indirectness	Imprecision	Publication Bias	Certainty
						downgrade.	
Behavioral Cooperation	1 RCT [30] (12)	Very serious (−2): universal; see text	N/A: single study	Serious (−1): narrow population	Serious (−1): small sample, explicitly underpowered; no significant differences on FBRS or VBRS	Not downgraded: Formal assessment not possible. Qualitative concerns present but insufficient to formally downgrade.	⨁◯◯◯ Very Low

* Critical outcome. † Potential participant overlap between studies. NS = not significant.
Certainty ratings: ⨁⨁⨁⨁ = high; ⨁⨁⨁◯ = moderate; ⨁⨁◯◯ = low; ⨁◯◯◯ = very low

Table S5. GRADE Evidence Profile: Ambient/Filtered Green Light vs. Other Wavelengths (Comparison B)

Outcome	No. of Studies (Participants)	Risk of Bias	Inconsistency	Indirectness	Imprecision	Publication Bias	Certainty
Pain	3 RCTs [27,32,35] + 2 NRSIs [38,39] (281†)	Very serious (−2): universal; see text	Serious (−1): green not consistently superior; green = blue > red; green vs. blue NS; relative perceptual phenomenon rather than therapeutic effect	Serious (−1): dental phobia, fibromyalgia, migraine photophobia, and retinal exam represent different pain constructs	Serious (−1): small samples; non-independent NRSI samples due to participant overlap	Not downgraded: Formal assessment not possible. Qualitative concerns present but insufficient to formally downgrade.	⨁◯◯◯ Very Low
Anxiety	1 RCT [32] (34)	Very serious (−2): universal; see text	N/A: single study	Serious (−1): fibromyalgia-related anxiety measured by PRO-	Serious (−1): pilot study; PROMIS finding from one of seven do-	Not downgraded: Formal assessment not possible. Qualitative con-	⨁◯◯◯ Very Low

Outcome	No. of Studies (Participants)	Risk of Bias	Inconsistency	Indirectness	Imprecision	Publication Bias	Certainty
				MIS in narrow population	mains without multiplicity correction	cerns present but insufficient to formally downgrade.	
Physiological Stress Markers	2 RCTs [27,28] (46)	Serious (−1): Instrument-derived, objective	Serious (−1): no significant sAA differences among green, blue, and red; green appeared to normalize HRV vs. blue but no formal interaction test	Serious (−1): dental phobia and migraine represent different stress response profiles	Serious (−1): small samples; neither powered for these comparisons	Not downgraded: Formal assessment not possible. Qualitative concerns present but insufficient to formally downgrade.	⨁◯◯◯ Very Low
Photophobia/Light Sensitivity	1 RCT [28] + 2 NRSIs [38,39] (120†)	Very serious (−2): universal; see text	Not serious: concordant (green least exacerbating) but non-independent; no normalization vs. blue	Serious (−1): laboratory perceptual paradigm rather than clinical treatment	Serious (−1): non-independent NRSI samples; small RCT sample	Not downgraded: Formal assessment not possible. Qualitative concerns present but insufficient to formally downgrade.	⨁◯◯◯ Very Low
Opioid Consumption	1 RCT [32] (34)	Serious (−1): Measurement objectivity	N/A: single study	Serious (−1): narrow population	Serious (−1): pilot study; not powered for definitive efficacy	Not downgraded: Formal assessment not possible. Qualitative concerns present but insufficient to formally downgrade.	⨁◯◯◯ Very Low

Outcome	No. of Studies (Participants)	Risk of Bias	Inconsistency	Indirectness	Imprecision	Publication Bias	Certainty
Patient Comfort/Cooperation	1 RCT [35] (100)	Serious (−1): unblinded design with non-validated subjective outcomes	N/A: single study	Serious (−1): narrow clinical context	Not serious: n = 100 with highly significant results	Not downgraded: Formal assessment not possible. Qualitative concerns present but insufficient to formally downgrade.	⊕◯◯◯ Very Low

* Critical outcome. † Potential participant overlap between studies. NS = not significant.

Certainty ratings: ⊕⊕⊕⊕ = high; ⊕⊕⊕◯ = moderate; ⊕⊕◯◯ = low; ⊕◯◯◯ = very low
